# ARPC1B is a novel prognostic biomarker for kidney renal clear cell carcinoma and correlates with immune infiltration

**DOI:** 10.3389/fmolb.2023.1202524

**Published:** 2023-09-19

**Authors:** Yong-Fei Tang, Bin Qiao, Ya-Bing Huang, Ming Wang

**Affiliations:** ^1^ Department of Pathology, Renmin Hospital of Wuhan University, Wuhan, China; ^2^ Department of Clinical Laboratory, Renmin Hospital of Wuhan University, Wuhan, China

**Keywords:** ARPC1B, Arp2/3 protein complex, kidney renal clear cell carcinoma, tumor immune microenvironment, immunotherapy

## Abstract

**Background:** Actin-related protein 2/3 complex subunit 1B (ARPC1B) is reported to be involved in tumorigenesis and progression. However, its role in kidney renal clear cell carcinoma (KIRC), correlation with tumor-infiltrating immune cells, and prognostic significance remain unclear.

**Methods:** Data sets from the TCGA, GTEx, GEPIA, GEO, UALCAN, and CPTAC databases were extracted and analyzed to investigate the expression difference, prognosis, and clinicopathological features of ARPC1B. Single-sample Gene Set Enrichment Analysis (ssGSEA), CIBERSORT, and TISCH2 analysis were used to examine the relationship between ARPC1B expression and tumor immune infiltration in KIRC. The potential function of ARPC1B in KIRC was explored by GO functional annotation and KEGG pathway analysis. The TIDE algorithm was used to predict and analyze the relationship between ARPC1B expression and response to immune checkpoint blockade (ICB). The expression of ARPC1B was further validated by using quantitative real-time polymerase chain reaction (qRT-PCR) and immunohistochemistry (IHC).

**Results:** The study showed that ARPC1B expression was an independent prognostic factor of KIRC, with high ARPC1B expression being associated with poor overall survival (OS). Enrichment of GO annotation and pathway analysis showed multiple immune-related functional pathways affected by ARPC1B such as regulation of immune effector process, inflammatory response regulation, antigen processing and presentation, asthma, autoimmune thyroid disease, graft versus host disease, intestinal immune network for IgA production, and type I diabetic mellitus. Moreover, ARPC1B expression positively correlated with infiltrating levels of myeloid-derived suppressor cells (MDSCs) and regulatory T cells (Tregs) in KIRC. Importantly, high ARPC1B expression predicted a low response to ICB in KIRC.

**Conclusion:** This study indicates that ARPC1B expression is an independent prognostic biomarker for OS in KIRC patients. High ARPC1B expression is closely associated with MDSCs and Tregs infiltration. These findings suggest that ARPC1B may serve as a biomarker for prognosis and immune infiltration in KIRC, potentially aiding in the development of novel treatment strategies to improve the survival outcomes for KIRC patients.

## Introduction

Renal cell carcinoma (RCC) is one of the most common and highly studied tumors in the genitourinary system. In the 2020 Global Cancer Statistics report, it was documented that renal cell carcinoma had a reported incidence of 431,288 cases globally. Within the same period, the mortality rate due to renal cell carcinoma was recorded at 179,368 deaths (Sung et al., 2021). Kidney renal clear cell carcinoma (KIRC) is a kind of tumor originating from the renal epithelium and accounts for approximately 70% of all renal cell carcinoma (RCC) cases (Choueiri and Motzer, 2017). It is distinguished by unique genetic and molecular alterations, diverse clinical courses, and potential specific therapeutic sensitivities (Argentiero et al., 2020). Nearly 30% of individuals diagnosed with KIRC are found to have distant metastases. Unfortunately, the 5-year overall survival rate of patients with metastatic KIRC is still less than 10% (Turajlic et al., 2018). Surgical treatment is currently regarded as the most effective method for treating KIRC patients (Porta et al., 2019). Regrettably, patients with metastases lose the opportunity for surgical treatment. In the treatment of advanced KIRC, commonly used drugs include tyrosine kinase inhibitors (TKIs) targeting the tumor cell growth pathway and vascular endothelial growth factor (VEGF) targeted therapies. Moreover, immune checkpoint blockade (ICB) therapy has emerged as a vital treatment modality for advanced KIRC, showing the ability to improve the overall survival rate of patients (Bahadoram et al., 2022). However, there are still some limitations and challenges that have to be addressed; for instance, only a portion of patients respond positively to ICB treatment, and there is the lack of effective predictive biomarkers and occurrence of immune-related adverse events (irAEs) (Ravi et al., 2020; Rini et al., 2021; Siddiqui et al., 2021). Therefore, it is imperative to explore more effective biomarkers for early diagnosis, treatment strategies, and prognosis prediction of KIRC.

Actin-related protein 2/3 complex subunit 1B (ARPC1B), a 41 kDa protein, is one of seven evolutionarily conserved subunits of the human Arp2/3 protein complex. The Arp2/3 protein complex forms a branched actin network between the cytoplasm and cell membrane and promotes cell movement. Diseases associated with mutations in the ARPC1B gene have been reported, which include immunodeficiency with inflammatory disease, congenital thrombocytopenia, and T-cell immunodeficiency (Somech et al., 2017; Volpi et al., 2019). Previous studies have shown that ARPC1B is involved in the development of various cancers, such as oral squamous cell carcinoma, hepatocellular carcinoma, and prostate cancer (Auzair et al., 2016; Huang et al., 2021; Gamallat et al., 2022). As an activator and substrate of Aurora kinase A (AURKA), ARPC1B knockout inhibits tumor migration and invasion by downregulating AURKA (Gamallat et al., 2022). The level of tumor infiltration lymphocytes (TILs) in the tumor microenvironment (TME) plays a critical role in the occurrence, development, metastasis, and treatment resistance of tumors. It has been reported that tumor-infiltrating lymphocytes, such as tumor-associated macrophages (TAMs) and myeloid-derived suppressor cells (MDSCs), participate in tumor immune escape and affect the efficacy of immunotherapy (Wu and Dai, 2017; Zhang et al., 2019). However, there is no report on the role of ARPC1B in tumor progression and tumor immunology in KIRC.

This study explores the expression pattern of ARPC1B and its prognostic value in KIRC. Our results suggest that ARPC1B may be a novel prognostic marker for KIRC, and its mechanism may be related to MDSCs and Tregs immunosuppressive cells. We conducted a preliminary exploration of the correlation between ARPC1B and immune cells, CD8^+^ T cells, and MDSCs. Immunohistochemistry was performed using antibodies against CD8 and CD33 to detect CD8^+^ T cells and MDSCs (Colbeck et al., 2017).

## Materials and methods

### Data collection and analysis of differential expressions

The expression, clinicopathological characteristics, and survival data of ARPC1B in KIRC were obtained from the UCSC Xena database. To assay the ARPC1B expression, tumor samples were obtained from the TCGA, and normal samples were obtained from both the TCGA and GTEx databases. GSE53757 and GSE66271 data sets were downloaded from the GEO DataSets, and paired *t*-test was used to analyze the difference in ARPC1B expression between tumor and normal tissues.

The Gene Expression Profiling Interactive Analysis (GEPIA) (http://gepia.cancer-pku.cn/index.html) is a publicly available web tool for gene expression analysis of tumor and normal samples based on the TCGA and GTEx databases. Here, GEPIA further confirmed the differential expression of ARPC1B in KIRC and normal tissues. In GEPIA, the data were analyzed using the ANOVA method, and thresholds of 0.01 for the *p*-value and 1 for fold change were set as the criteria for determining the statistical significance of the results. The Clinical Proteomic Tumor Analysis Consortium (CPTAC) integrates genomic and proteomic data to identify and describe all proteins in tumor and normal tissues, thereby helping to discover candidate proteins that can be used as tumor biomarkers.

### UALCAN database analysis

The UALCAN (http://ualcan.path.uab.edu/index.html) is a comprehensive interactive network resource for analyzing cancer Omics data. It enables researchers to collect valuable information and data on the genes that they are interested in (Chandrashekar et al., 2017). In this study, the clinical characteristics and total protein expression of KIRC were analyzed by using UALCAN. In UALCAN, various statistical methods were utilized to analyze the data.

### cBioPortal database analysis

The cBioPortal (http://cbioportal.org) is a database that provides visualization and analysis of cancer genomics data sets, which include copy number aberrations and mutations. The portal provides access to data from 147 individual cancer research projects of 31 cancer types and over 21,000 samples (Gao et al., 2013). We analyzed the cBioPortal to explore gene copy number variation and mutation of ARPC1B in KIRC. In the cBioPortal, we started by searching for the KIRC data set by using the search bar. Then, within the “genetic alterations” or “genes” sections, we typed “ARPC1B” in the search box to find the ARPC1B gene.

### Identification of prognostic factors for OS in KIRC

Univariate and multivariate Cox regression analyses were used to evaluate APRC1B, and five more major clinical and prognostic factors, namely, age, race, gender, grading, and stage, were used to determine the proper terms for establishing the nomogram. The forest was used to show the *p*-value, HR, and 95% CI of each variable through the “forest plot” R package. A nomogram was developed on the basis of the results of the multivariate Cox proportional hazards analysis to predict the X-year overall recurrence.

### TISIDB database analysis

The TISIDB database (http://cis.hku.hk/TISIDB) is a web portal for tumor and immune system interaction, which integrates multiple heterogeneous data types. By retrieving various data resources stored in TISIDB, users can easily find the immune relationship between specific genes and tumor occurrence environments (Ru et al., 2019). In this study, we used TISIDB to explore the association between ARPC1B with immune subtypes, molecular subtypes, and drug targets. After inputting “ARPC1B” into the search bar of the TISIDB and pressing the search button, the information and functions related to the ARPC1B gene were displayed.

### CIBERSORT and ssGSEA

CIBERSORT was used to investigate the difference in immune cell subtypes. According to the expression profile, the proportion of 22 infiltrating immune cells in the KIRC samples was counted by the R package “CIBERSORT” (Newman et al., 2015). The single-sample Gene Set Enrichment Analysis (ssGSEA) was used to explore the abundance of 29 infiltrating immune cells and immune-related functions in KIRC tissues by using the R package “GSVA” in the KIRC expression profile (Hänzelmann et al., 2013). Then, the R packages “ggplot2,” “ggpubr,” and “ggExtra” were used to analyze the association between ARPC1B and immune cell infiltration.

### TISCH2 and Tabula Muris database analysis

Tumor Immune Single-Cell Hub 2 (TISCH2) provides detailed annotations of immune cell types at the single-cell level, which can help users explore the tumor microenvironment of different cancers. The Tabula Muris is a collection of mouse single-cell transcriptome databases, which allows the comparative analysis of gene expression between different cell types. In this study, we used the TISCH2 database to explore the association between ARPC1B and immune cells. The Tabula Muris database was used to locate the expression site of ARPC1B in normal renal tissues.

### Specimen source and pathological examination

Human kidney renal clear cell carcinoma specimens were obtained from the Renmin Hospital of Wuhan University. All patients signed an informed consent form, which was approved by the Clinical Research Ethics Committee of the Renmin Hospital of Wuhan University. The results of surgical resection and immunohistochemistry (IHC) were read and finally diagnosed by two senior pathologists in Renmin Hospital of Wuhan University.

### Quantitative real-time polymerase chain reaction

Total RNA was extracted by using TRIzol Reagent (Invitrogen, United States) according to the manufacturer’s protocol. cDNA was obtained by the reverse transcription system. Quantitative real-time polymerase chain reaction (qRT-PCR) was performed using SYBR Green I fluorescent dye (TaKaRa Bio, China) on the LightCycler^®^ 480 Instrument II (Roche, Germany). The relative expression of ARPC1B in each sample was normalized to GAPDH and calculated by using the 2^−ΔΔCt^ method. The primers were described as follows: ARPC1B, gttat ttcga gcagg agaat gac (F), gtagg ctgaa aagat ccgac a (R); GAPDH, gtgga cctga cctgc cgtct (F), ggagg agtgg gtgtc gctgt (R).

### Immunohistochemistry

The immunohistochemistry (IHC) stain was performed on the Dako EnVision detection system. The formalin-fixed paraffin-embedded (FFPE) sections (3 μm thick) were dewaxed, hydrated, and then incubated in Tris/EDTA buffer (pH 9.0) for antigen retrieval. Subsequently, endogenous peroxidase was blocked with 3% hydrogen peroxide for 10 min. Then, the FFPE sections were incubated with ARPC1B rabbit polyclonal antibody (1:500, Bioss Biotechnology, China), CD8 monoclonal antibody (working solution, Agilent Dako, Denmark), and CD33 rabbit polyclonal antibody (1:400, Bioss Biotechnology, China), followed by incubation with HRP secondary antibody, and finally stained with peroxidase/DAB immunohistochemistry.

### Gene Set Enrichment Analysis

The GSEA was performed using the GSEA 4.1.0 software to explore the potential biological mechanism of ARPC1B mRNA expression levels affecting the development of KIRC (Subramanian et al., 2005). The c2.cp.kegg.v7.4.symbols.gmt data set was obtained from the Molecular Signatures Database (MsigDB) as the reference. The enrichment analysis was repeated 1,000 times at a time, and a false discovery rate (FDR, *q*-value) < 0.05 was used to identify the significantly enriched pathways.

### Immune checkpoint analysis

Using the online TIMER (https://cistrome.shinyapps.io/timer/) database, we explored the correlation between ARPC1B mRNA expression and the genes SIGLEC15, IDO1, cytotoxic T lymphocyte antigen 4 (CTLA4), CD274, HAVCR2, LAG3, PDCD1, and PDCD1LG2 selected from the immune checkpoints. The TIDE model was used to predict immune potential checkpoint blockade response by using a tumor sample gene expression profile. It integrated the expression signatures of T-cell dysfunction and T-cell exclusion to evaluate tumor immune escape (Jiang et al., 2018). We uploaded the standardized expression data to the TIDE (http://tide.dfci.harvard.edu/) website and downloaded the analysis results to explore the relationship between the ARPC1B mRNA expression and immunotherapy responder, TIDE score, and immune dysfunction.

### Statistical analysis

The *t*-test was applied to analyze the difference between groups for variables with a normal distribution. Otherwise, the Mann–Whitney *U* test was applied. The receiver operating characteristic (ROC) curve was used to evaluate the diagnostic value of the ARPC1B gene expression, with the area under the curve (AUC) used as the diagnostic value. The Cox proportional hazards model, Kaplan–Meier analysis, and the log-rank test were performed for all survival analyses. The Spearman’s test or Pearson’s test was performed to examine the correlations between two variables. All statistical tests were two sided, and a *p*-value < 0.05 was considered statistically significant. The R software (version 4.0.2) was used for statistical analyses.

## Results

### Different expressions of ARPC1B in KIRC and normal tissues

**FIGURE 1:**
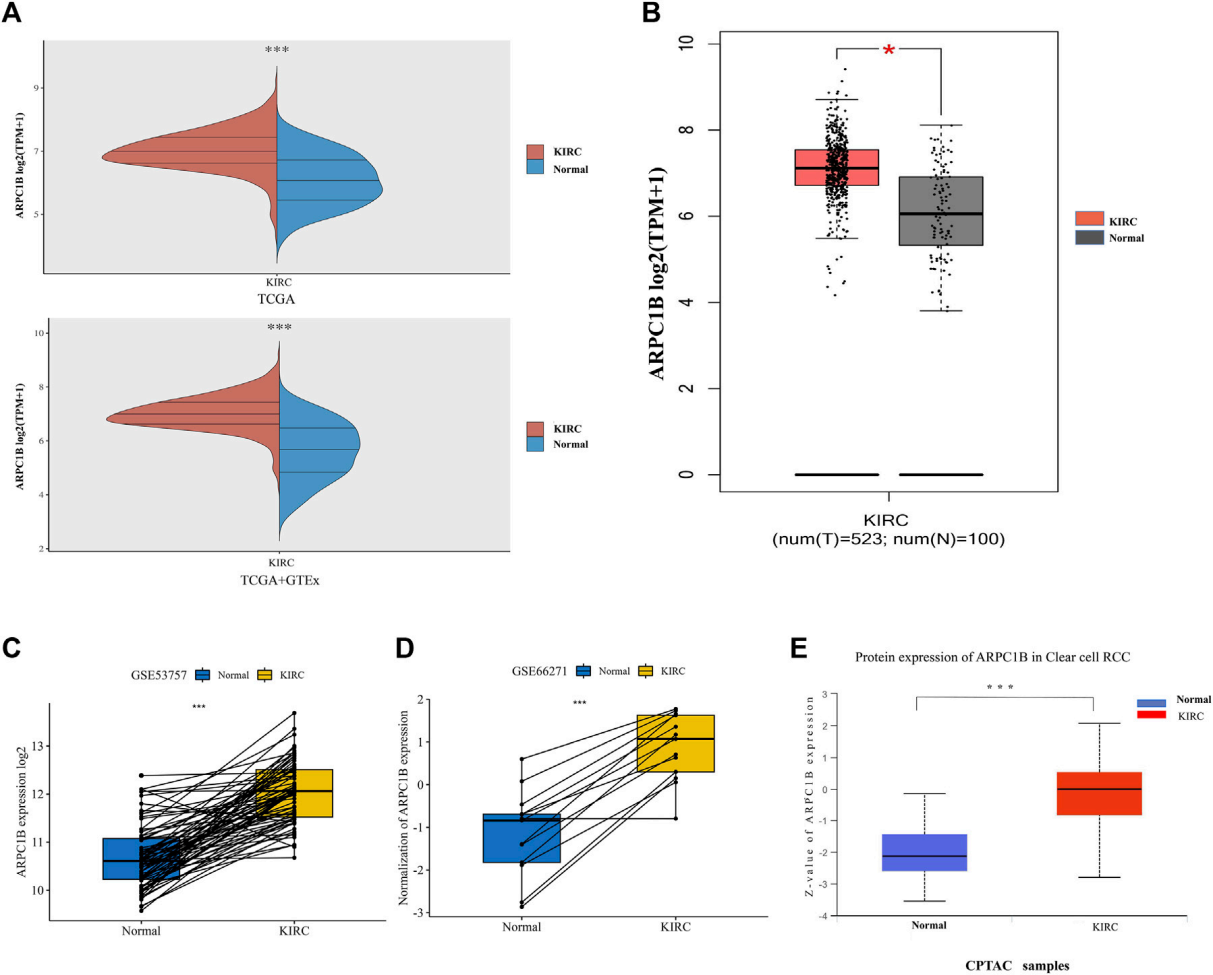
Different mRNA and protein expressions of ARPC1B in KIRC and normal tissues. **(A)** ARPC1B mRNA expression levels in KIRC and normal tissues from TCGA and GTEx databases. **(B)** ARPC1B mRNA expression levels in KIRC and normal tissues in the GEPIA database. **(C and D)** Differential ARPC1B expression levels in KIRC and normal tissues from the GSE53757 and GSE66271 data sets. **(E)** The different total ARPC1B protein expression in KIRC and normal tissues from the CPTAC database. *p < 0.05, **p < 0.01, ***p < 0.001.

The mRNA expression of ARPC1B between KIRC and normal tissues was compared using the TCGA database. Data (which included 532 KIRC samples and 72 normal samples) of mRNA expression were obtained. The data from the TCGA normal samples and GTEx samples were merged as normal groups for further analysis with TCGA tumor samples (Figure 1A). It was found that the mRNA expression of ARPC1B in KIRC was significantly higher than that in normal tissues (*p* < 0.001). Next, the GEPIA database was used to validate the results (Figure 1B). A paired *t*-test was performed on the data of GSE53757 and GSE66271 of KIRC and paired adjacent normal tissues for differential mRNA expression analysis (Figures 1C, D). The total ARPC1B protein expression in KIRC was significantly higher than that in normal tissues from the CPTAC database (Figure 1E).

### Role of ARPC1B expression in clinical characteristics of KIRC

**FIGURE 2:**
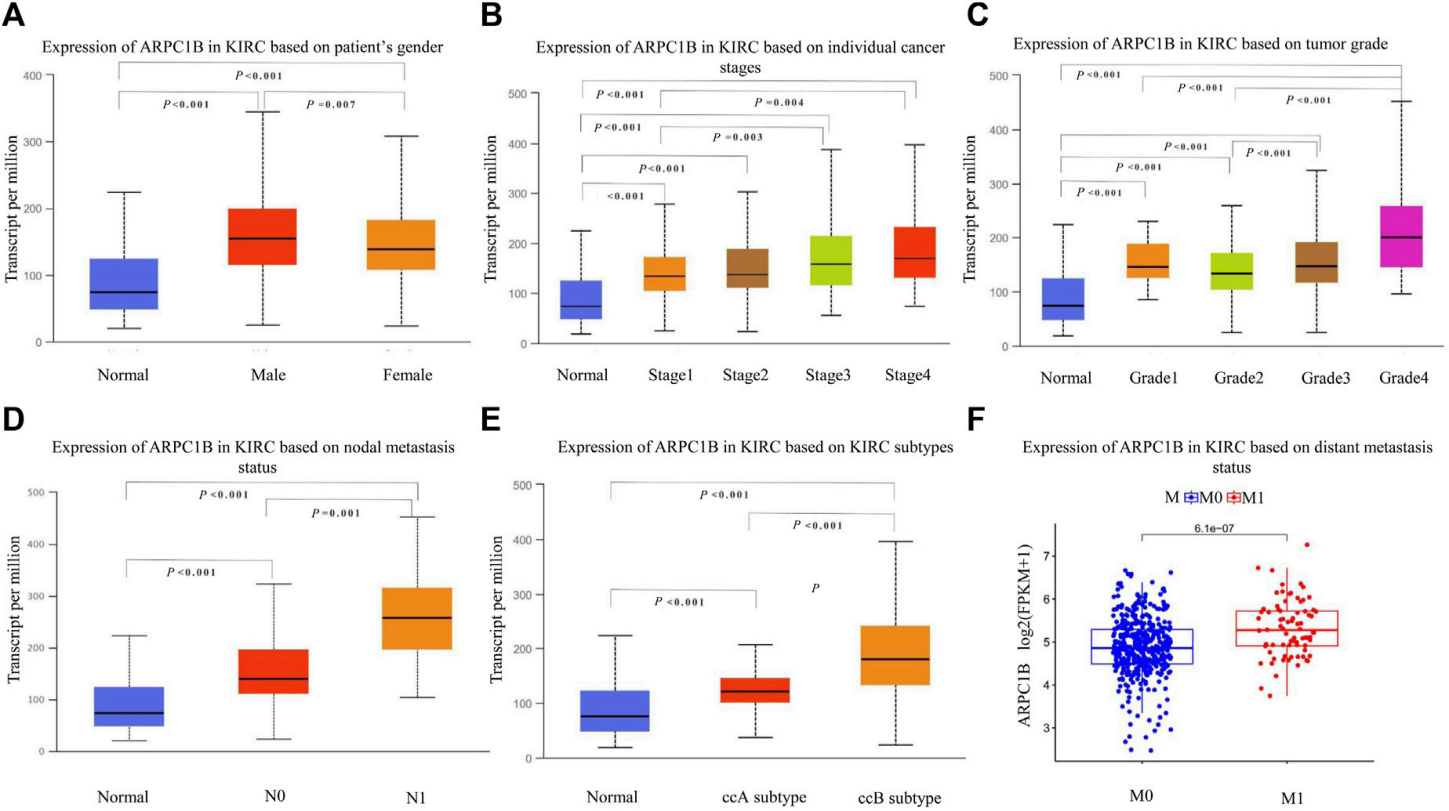
Relationship between ARPC1B expression and clinicopathological characteristics in KIRC. **(A–C)** Relative mRNA expressions of ARPC1B in relation to gender, tumor stage, and tumor grade status. **(D–F)** Relative mRNA expressions of ARPC1B with respect to node metastasis, subtypes, and distant metastasis status.

We used the UALCAN database to investigate the expression of ARPC1B in different clinical features of KIRC. The results showed that ARPC1B was significantly differentially expressed depending on the patient’s gender, different cancer stages, different tumor grades, nodal metastasis status, and histological subtypes of KIRC. Using the downloaded TCGA data for analysis, it was found that the expression of ARPC1B was significantly different in the different distant metastasis statuses of KIRC (Figures 2A–F). The expression of ARPC1B increased with increase in staging and grading, lymph node metastasis, and distant metastasis.

### Diagnostic value of ARPC1B expression in KIRC

**FIGURE 3:**
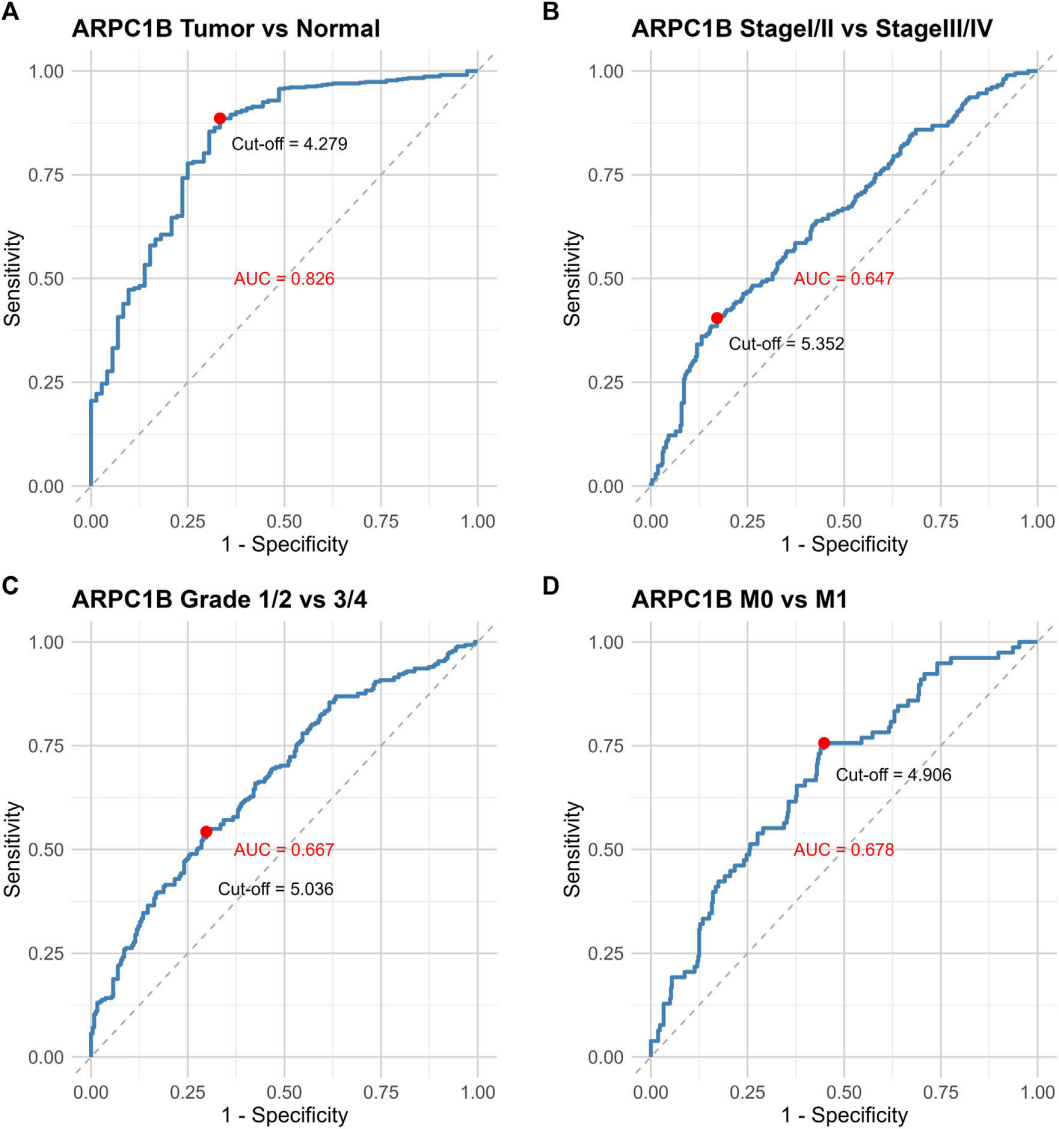
ROC curves illustrating the performance of ARPC1B expression in different scenarios. **(A)** ROC curves of ARPC1B expression in KIRC and normal tissues. **(B)** ROC curves comparing stage Ⅰ/Ⅱ with stage Ⅲ/Ⅳ. **(C)** ROC curves distinguishing between grade 1/2 and grade 3/4. **(D)** ROC curves for distinguishing metastases M0 and M1.

The ROC curve was used to evaluate the differential value of ARPC1B expression in KIRC and normal tissues, stage I/II and stage III/IV, grade 1/2 and grade 3/4, and metastases M0 and M1. Their AUCs were 0.826, 0.647, 0.667, and 0.678, respectively (Figures 3A–D). Among them, the maximum AUC value was the expression of ARPC1B that was used to distinguish cancerous from normal tissues. The optimal cutoff value was 4.279, and the specificity and sensitivity were 66.7% and 88.6%, respectively.

### Genomic alterations of ARPC1B in KIRC

**FIGURE 4:**
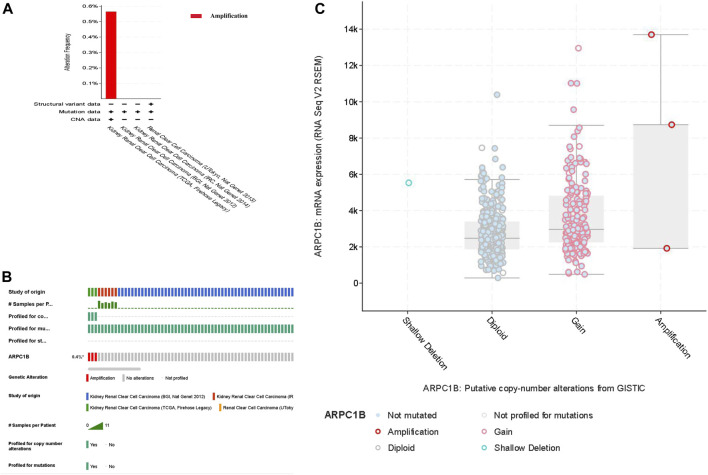
Analysis of genomic alterations of ARPC1B in KIRC. **(A)** Genomic alterations of ARPC1B and the corresponding data set. **(B)** Types of ARPC1B gene alterations and their incidence. **(C)** Relationship between copy number variation and expression levels of ARPC1B.

We selected four databases comprising a total of 821 patients. All four data sets detected mutations, with only the TCGA data identifying copy number variations (CNVs) and only the UTokyo data detecting structural variants. The results showed that three patients, approximately 0.4% of the tested population, exhibited genetic alterations in ARPC1B. The type of ARPC1B gene alterations in KIRC was copy number variations (CNVs), resulting in changes in gene expression. The ARPC1B expression appeared to increase as the number of copies increased (Figures 4A–C).

### Prognostic value of ARPC1B in KIRC

**FIGURE 5:**
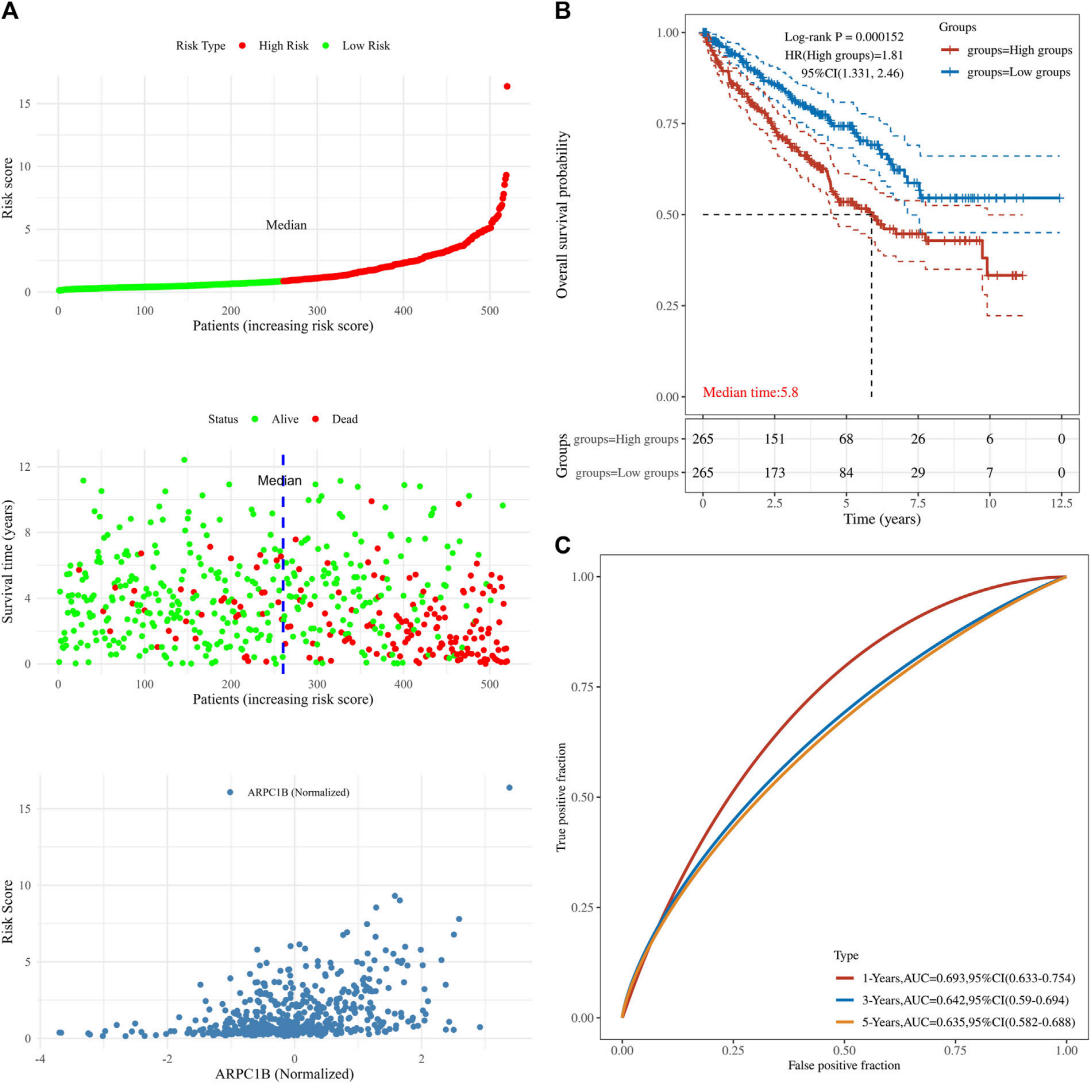
Associations between ARPC1B expression and patient survival based on the TCGA data set. **(A)** The top chart displays patients sorted by risk scores and divided into high-risk and low-risk groups based on the median number of patients. The middle plot shows the relationship between survival time, survival state, and the high-/low-risk groups. The bottom plot depicts the relationship between risk scores and normalized expression levels of ARPC1B. **(B)** The Kaplan–Meier survival analysis of ARPC1B expression, with comparisons among different groups made using the log-rank test. HR represents the hazard ratio of high-expression samples relative to low-expression samples. HR > 1 indicates that the gene is a risk factor, while HR < 1 indicates that the gene is a protective factor. HR (95% CI) represents the hazard ratio along with its corresponding 95% confidence interval, as well as the median survival time (LT50) for different groups. **(C)** The ROC curve of the ARPC1B expression. The higher values of AUC correspond to a higher predictive power.

This analysis was instrumental in elucidating the complete panorama of the correlation between ARPC1B expression, survival time, survival status, and risk scores within the TCGA data set. It aided in providing a comprehensive understanding of their interrelationship. In Figure 5, the top chart displays patients sorted by risk scores and divided into high-risk and low-risk groups based on the median number of patients. The middle chart illustrates the relationship between survival time, survival status, and the high-risk/low-risk groups, indicating a higher number of deaths in the high-risk group. The bottom chart demonstrates the increased standardized expression levels of ARPC1B with higher risk scores (Figure 5A). The Kaplan–Meier survival analysis was used to evaluate the relationships between ARPC1B expression and patient survival. The results showed that high ARPC1B expression was significantly associated with poor OS in KIRC patients (Figure 5B, HR = 1.81, *p* < 0.001). We performed the survival area under the curve (AUC) analysis to evaluate the predictive ability of ARPC1B. As shown in Figure 5C, the 1-year AUC was 0.693 with a 95% CI (0.633–0.754), the 3-year AUC was 0.642 with a 95% CI (0.59–0.694), and the 5-year AUC was 0.635 with a 95% CI (0.582–0.688).

**FIGURE 6:**
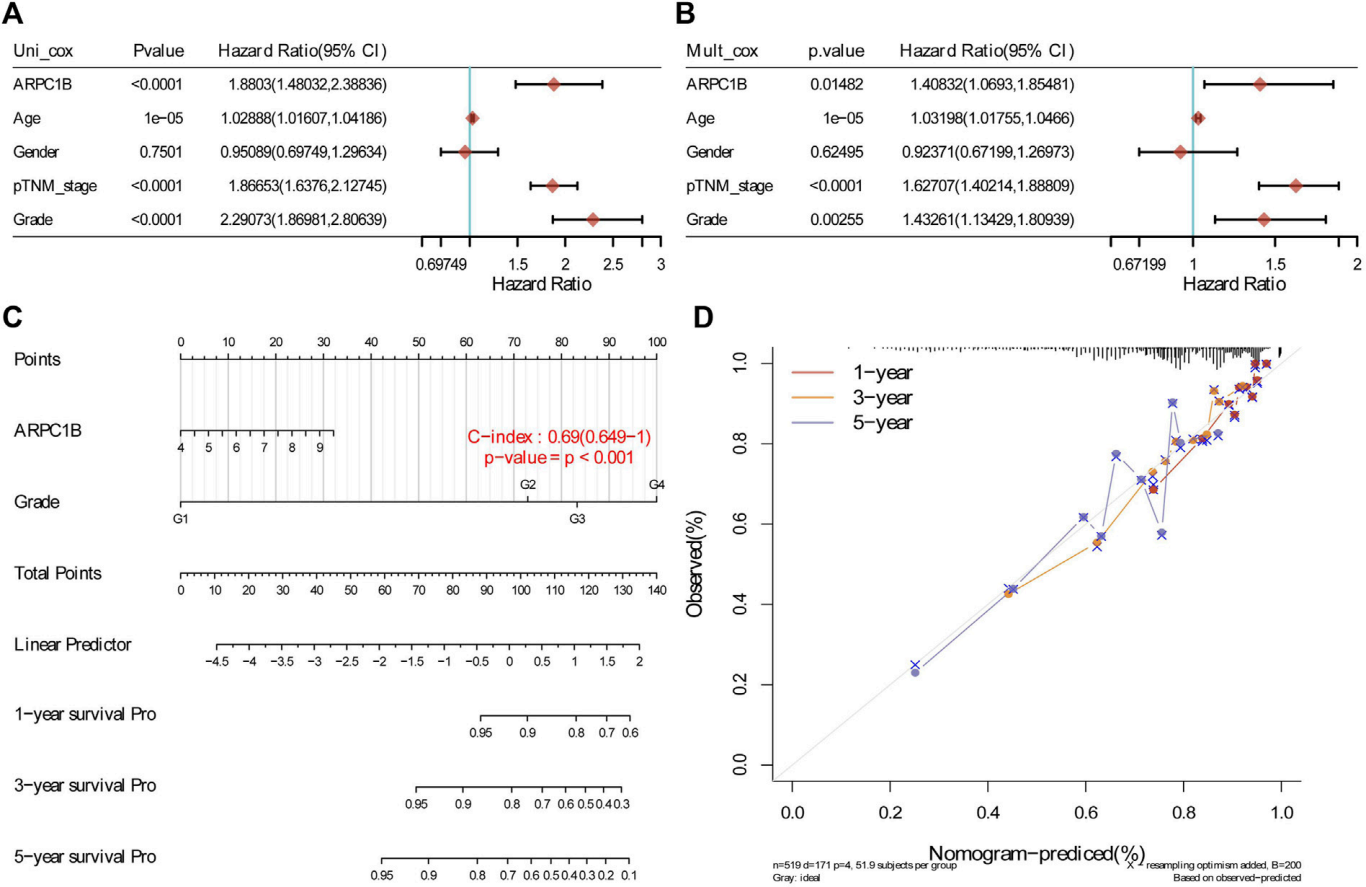
Construction and evaluation of a prognostic model using the expression of ARPC1B and other clinical indicators in KIRC. **(A)** Univariate Cox regression analysis was performed to assess the statistical significance of ARPC1B and other clinical indicators, displaying the corresponding *p*-value, HR, and 95% CI. **(B)** Multivariate Cox regression analysis was conducted to further elucidate the prognostic significance of ARPC1B and the selected clinical indicators. **(C)** Nomograms were developed to predict the 1-, 3-, and 5-year overall survival of KIRC patients based on the expression of ARPC1B and additional factors. **(D)** Calibration curve illustrating the performance of the overall survival nomogram model in the discovery group. The dashed diagonal line represents the ideal nomogram, while the blue, red, and orange lines represent the observed nomogram’s predicted survival rates at 1-, 3-, and 5-year intervals.

The results of the univariate analysis indicated that ARPC1B (HR = 1.8803, *p* < 0.0001), age (HR = 1.02888, *p* < 0.0001), stage (HR = 1.86653, *p* < 0.0001), and grade (HR = 2.29073, *p* < 0.0001) had a prognostic value for the OS of KIRC. Through multivariable analysis, the results showed that ARPC1B (HR = 1.40832, *p* = 0.0148), age (HR = 1.03198, *p* < 0.0001), stage (HR = 1.62707, *p* < 0.0001), and grade (HR = 1.43261, *p* = 0.0025) had a prognostic value for the OS of KIRC. These results suggest that ARPC1B expression was an independent prognostic factor for KIRC. Then, ARPC1B, age, and grade were selected for establishing the OS and plotted in the nomogram. The nomogram that predicted 1-, 3-, and 5-year OS is shown in Figure 6.

### Association between ARPC1B expression and tumor immune infiltration in KIRC

**FIGURE 7:**
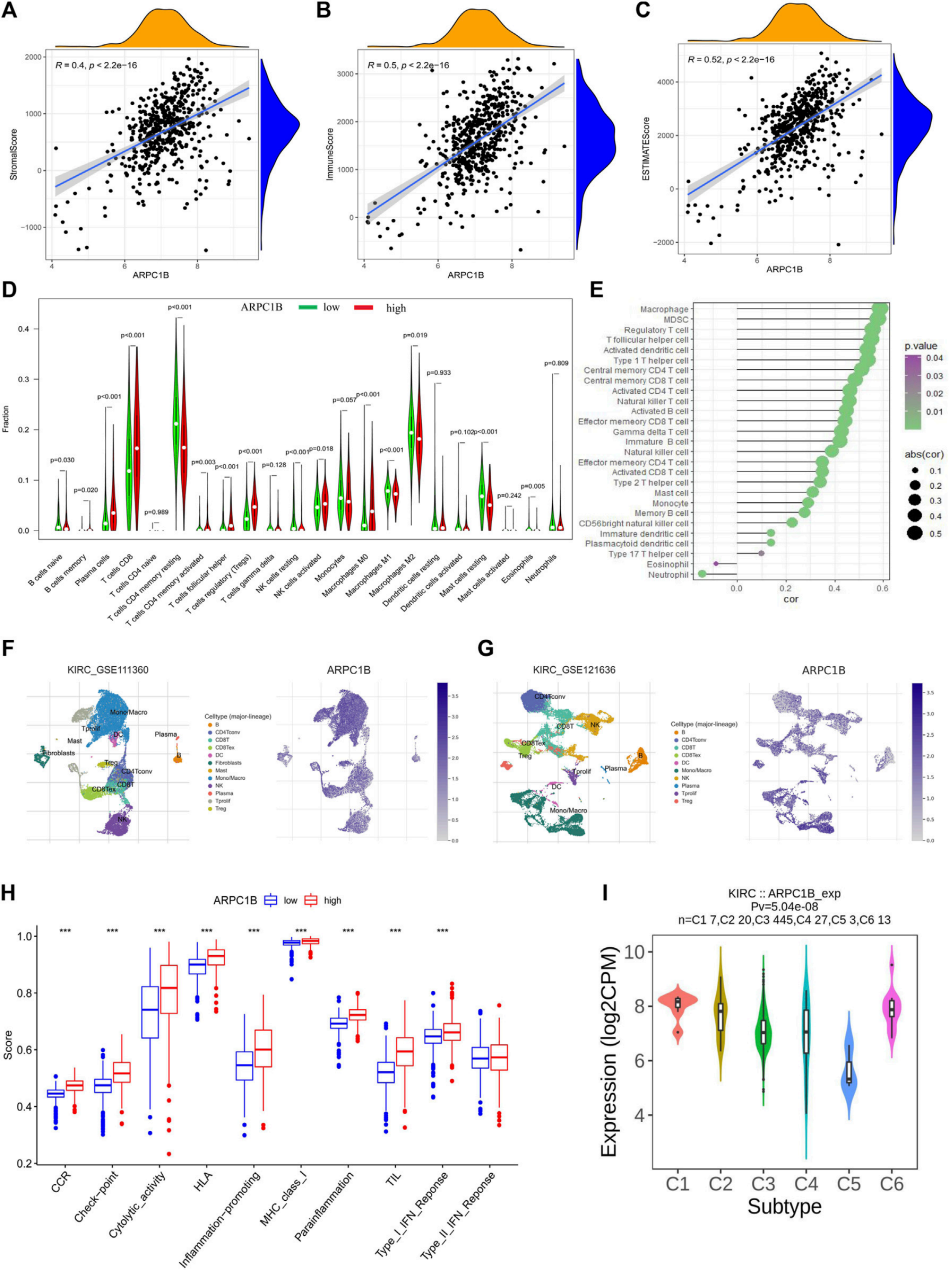
Correlations between ARPC1B expression and tumor microenvironment, which include immune cell infiltration, immune function, and immune subtypes in the KIRC. **(A–C)** The stromal score **(A)**, immune score **(B)**, and estimate score **(C)** were analyzed for their correlation with ARPC1B expression. The immune infiltration landscape was assessed using the CIBERSORT method **(D)**, while ssGSEA analysis was employed to analyze immune cell infiltration in KIRC samples from the TCGA data set **(E)**. **(F and G)** TISCH2 analysis reveals the correlation between ARPC1B expression and immune cell populations. **(H)** ssGSEA analysis utilized to examine immune function. **(I)** Investigated relationship between ARPC1B expression and immune subtypes.

The stromal score, immune score, and estimate score of KIRC were investigated by the ESTIMATE algorithm (Wu et al., 2021), and the correlation between the ARPC1B expressing level and these three scores was analyzed. The results showed that the expression of ARPC1B had a significant positive correlation with stromal score (Figure 7A, *R* = 0.40, *p* < 2.2e-16), immune score (Figure 7B, *R* = 0.50, *p* < 2.2e-16), and estimate score (Figure 7C, *R* = 0.52, *p* < 2.2e-16) in KIRC.

The CIBERSORT algorithm was applied to calculate the relative proportions of the infiltration of 22 immune cell subtypes in KIRC tissue. As shown in Figure 7D, the proportion of immune cells in KIRC tissues with high ARPC1B expression was significantly different from that with low ARPC1B expression. The results revealed that memory B cells, plasma cells, CD8^+^ T cells, CD4^+^ memory-activated T cells (CD4^+^ Tma), follicular helper T cells (Tfh), Tregs (regulatory T cells), activated natural killer cells (NKa), and M0 macrophages had a higher proportion in the high ARPC1B expression than in the low ARPC1B expression. Meanwhile, CD4^+^ resting memory T cells (CD4^+^ Tmr), resting natural killer cells (NKr), M1 macrophages (M1), M2 macrophages (M2), resting mast cells (Mr), activated mast cells (Ma), and eosinophils had a lower proportion in the high ARPC1B expression than in the low ARPC1B expression.

The ssGSEA was implemented to analyze the correlation between ARPC1B expression and immune cell infiltration using the TCGA data. The results indicated that ARPC1B expression was positively correlation with the filtrating levels of macrophages, MDSCs, Tregs, Tfh, activated dendritic cells (DCa), immature dendritic cells (DCi), plasmacytoid dendritic cells (DCp), type-1 T helper cells (Th1), type-2 T helper cells (Th2), type-17 T helper cells (Th17), central memory CD4^+^ T cells (CD4^+^ Tcm), central memory CD8^+^ T cells (CD8^+^ Tcm), activated CD4^+^ T cells (CD4^+^ Ta), activated CD8^+^ T cells (CD8^+^ Ta), effector memory CD4^+^ T cells (CD4^+^ Tem), effector memory CD8^+^ T cells (CD8^+^ Tem), gamma delta T cells (γδT), memory B cells (Bm), immature B cells (Bi), natural killer cells (NK), CD56 bright natural killer cells (CD56^+^ NK), mast cells, and monocytes, while it was negatively correlated with the infiltration of eosinophils and neutrophils (Figure 7E).

The results of the analysis of the KIRC_GSE111360 and KIRC_GSE121636 data sets from TISCH2 revealed that memory B cells, plasma cells, CD8^+^ T cells, CD4^+^ memory, CD4^+^ Tconv, Tregs, Tprolif, CD8^+^ T, the exhausted CD8^+^ T cell (CD8^+^ Tex), NK, B, DC, and monocytes/macrophages were positively correlated with ARPC1B expression (Figures 7F–G, Supplementary Figure S1).

In summary, the main immune cells that were positively correlated with ARPC1B expression are M0 macrophages, MDSCs, Tregs, Tfh, CD8^+^ T cells, CD8^+^ Tex, CD4^+^ T cells, Bm, NKa, CD56^+^ NK, and plasma cells. The main immune cells that were negatively correlated with ARPC1B expression are M1, M2, eosinophils, and neutrophils.

### Association between ARPC1B expression and immune function in KIRC

We employed the ssGSEA method from the gsav package to evaluate the immune function score for each sample in KIRC. The *t*-test was conducted to assess the disparity in immune function scores between the ARPC1B low- and high-expression groups.

The findings demonstrated a statistically significant increase in CCR, checkpoint, cytolytic activity, HLA, inflammation promotion, MHC class I, para-inflammation, TIL, and type I IFN responses in the ARPC1B high-expression group when compared to the low-expression group (Figure 7H). The high expression of ARPC1B correlates with increased immune/inflammation activity, but it is associated with a worse prognosis, suggesting the occurrence of immune escape.

### ARPC1B expression related to immune and molecular subtypes in KIRC

The TISIDB website was utilized to analyze the role of ARPC1B in immune and molecular subtypes of KIRC. The immune subtypes were categorized into six types: C1 (wound healing), C2 (IFN-γ), C3 (inflammation), C4 (lymphocyte depletion), C5 (immune quiet), and C6 (TGF-b). The findings demonstrated that ARPC1B exhibited high expression in the C1 and C6 subtypes while showing low expression in the C5 subtype (Figure 7I).

### GO functional annotations and KEGG pathway analysis

**FIGURE 8:**
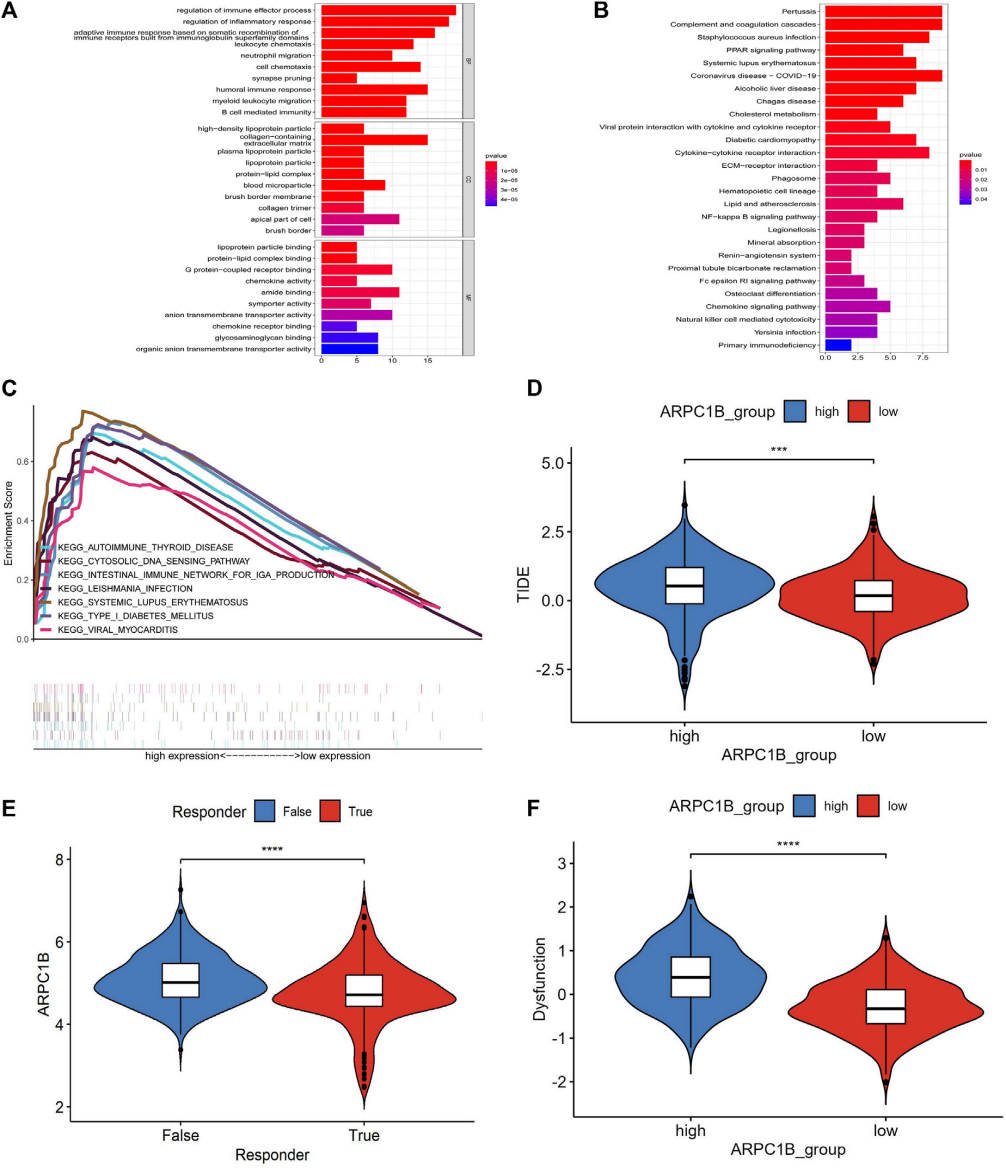
Results of GO function annotation and KEGG path enrichment analysis, as well as responses to immune checkpoint blockade and their association with ARPC1B expression. **(A)** Enrichment analysis for GO function annotations. **(B)** Enrichment analysis for KEGG pathways based on ARPC1B expression. **(C)** GSEA analysis reveals the activation of signaling pathways associated with high APRC1B expression in KIRC. **(D)** Variation in TIDE scores between high and low APRC1B expression groups. **(E)** Differential responses to immune checkpoint blockade based on ARPC1B expression in KIRC. **(F)** Variation in T-cell dysfunction between high and low APRC1B expression groups. *p < 0.05, **p < 0.01, ***p < 0.001, ****p < 0.0001.

We used the GO functional annotations and KEGG pathway analysis to investigate the potential action mechanism of ARPC1B in KIRC. The GO functional annotations revealed enrichment in several biological processes, such as in the regulation of immune effector process, regulation of inflammatory response, adaptive immune response based on somatic recombination of immune receptors, leukocyte chemotaxis, neutrophil migration, cell chemotaxis, synapse pruning, humoral immune response, humoral immune response, myeloid leukocyte migration, and B-cell–mediated immunity (Figure 8A).

In the KEGG pathway analysis, the enriched biological processes were associated with pertussis, complement and coagulation cascades, *Staphylococcus aureus* infection, PPAR signaling pathway, systemic lupus erythematosus, coronavirus disease, alcoholic liver disease, Chagas disease, cholesterol metabolism, viral protein interaction with cytokines and cytokine receptors, diabetic cardiomyopathy, and cytokine–cytokine receptor interaction (Figure 8B).

### Gene set enrichment analysis

To identify the differential activation signaling pathways in KIRC, we conducted the GSEA analysis using data sets with low and high ARPC1B expressions. The analysis showed that 99 of the 177 gene sets were upregulated in the high ARPC1B expression phenotype, and 11 gene sets were significantly enriched at nominal *p*-value < 1%, nominal *p* < 0.05, NES > 1.0, and FDR *q* < 0.25. In the low ARPC1B expression phenotype, 78 of the 177 gene sets were upregulated, and only 1 gene set was significantly enriched at nominal *p*-value < 1%, nominal *p* < 0.05, NES > 1.0, and FDR *q* < 0.25.

The differentially enriched gene sets in the high ARPC1B expression phenotype were found to be associated with several immunoregulatory genes, which included those involved in antigen processing and presentation, asthma, autoimmune thyroid disease, cytosolic DNA sensor pathway, graft versus host disease, intestinal immune network for IgA production, Leishmania infection, system lupus erythematosus, and type I diabetic mellitus (Figure 8C).

### Immune checkpoint analysis

The SIGLEC15, IDO1, CTLA4, CD274, HAVCR2, LAG3, PDCD1, and PDCD1LG2 from the immune checkpoint genes were selected. The result illustrated a positive correlation between ARPC1B expression and SIGLEC15 (*R* = 0.353, *p* = 4.82e-17), CTLA4 (*R* = 0.385, *p* = 2.57e-20), HAVCR2 (*R* = 0.149, *p* = 0.0005), LAG3 (*R* = 0.489, *p* = 1.78e-30), PDCD1 (*R* = 0.48, *p* = 4.21e-32), and PDCD1LG2 (*R* = 0.375, *p* = 3.18e-19). There was no correlation between ARPC1B expression with IDO1 (*R* = −0.041, *p* = 0.345) and CD274 (*R* = 0.015, *p* = 0.729).

The TIDE algorithm was used to predict immunotherapy response. Patients with a high TIDE score have a lower probability of reactivity to ICB and demonstrate poor treatment efficacy. The TIDE score of the low-expression group of ARPC1B was lower than that of the high-expression group (Figure 8D, *p* < 0.001), indicating that patients in the low-expression group may exhibit better responses to ICB treatment (Figure 8E, *p* < 0.0001). Additionally, the TIDE algorithm found that the T-cell dysfunction scores were higher in the high-expression group of ARPC1B than in the low-expression group (Figure 8F, *p* < 0.0001).

### Possible drugs targeting ARPC1B

We used the TISIDB website, which provides access to comprehensive data from the DrugBank database, to identify drugs that target ARPC1B. Within the database, we discovered two small-molecule drugs associated with ARPC1B. Our search yielded two small-molecule drugs available in the database. The first drug, assigned with the DrugBank access number DB08235, is named N-[2-(2-methyl-1H-indole-3-yl)ethyl]thiophene-2-carboxamide. The second drug, identified as DB08236, is referred to as (2S)-2-(3-bromophenyl)-3-(5-chloro-2-hydroxyphenyl)-1,3-thiazolidine-4-one. Both of these drugs have been developed to specifically target ARPC1B.

### Validation of ARPC1B mRNA expression in KIRC

**FIGURE 9:**
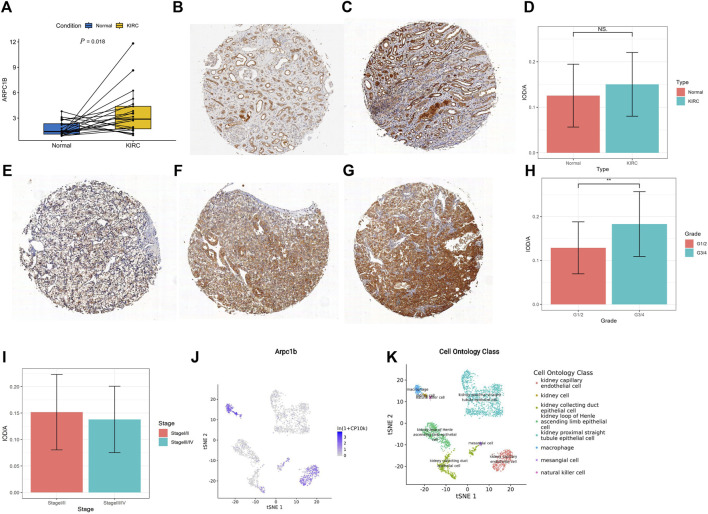
Expression of ARPC1B in normal and KIRC tissues. **(A)** qRT-PCR analysis comparing ARPC1B expression between KIRC and normal tissues. **(B–C)** IHC images demonstrating ARPC1B expression in normal renal tissue (weak and strong expressions). **(E–G)** IHC images depicting ARPC1B expression in KIRC with weak, medium, and strong expressions, respectively. The ratio of IOD to the area of IHC images is shown for comparisons between KIRC and normal tissues **(D)**, grade 1/2 versus grade 3/4 **(H)**, and stage Ⅰ/Ⅱ versus stage Ⅲ/Ⅳ **(I)**. **(J–K)** ARPC1B expression in cell ontology classes using the Tabula Muris database. NS (not significant with a *p*-value greater than 0.05), *p < 0.05, **p < 0.01, ***p < 0.001.

A total of 21 pairs of KIRC tissues and adjacent normal tissues were analyzed using qRT-PCR to validate the changes in ARPC1B mRNA. The results revealed a significantly higher expression level of ARPC1B mRNA in KIRC tissues than in normal tissues (Figure 9A, *p* = 0.018).

The density of immunohistochemical staining signals was evaluated using the Image-Pro Plus 6.0 image analysis software (Media Cybernetics, United States). The average optical density of ARPC1B immunohistochemical staining was determined for 53 KIRC tissues (0.14528 ± 0.07009) and nine normal tissues (0.12558 ± 0.06907). The cytoplasmic localization of ARPC1B immunohistochemical staining was observed in both normal kidneys (Figures 9B, C) and KIRC (Figures 9E–G). The results indicated no significant difference in ARPC1B expression between normal and KIRC tissues (Figure 9D, *p >* 0.05). In normal tissues, ARPC1B showed specific expression in renal tubular cells, with varying levels of expression intensity, spanning from weak to strong. Interestingly, it was observed that strongly expressed ARPC1B was often associated with degenerative or aging renal tubular cells. The expression intensity of ARPC1B in grade 3/4 was higher than that in grade 1/2 (Figure 9H, *p <* 0.01). Unfortunately, due to the small number of advanced KIRC cases, statistical analysis cannot be performed to compare stage I/II and stage III/IV (Figure 9I). The Tabula Muris database showed that the expression of ARPC1B was closely related to renal cells, macrophages, leukocytes, and kidney capillary endothelial cells by the droplet method (Figures 9J, K, Supplementary Figure S2).

### Relationship between CD8^+^ T cells and MDSCs, and expression of ARPC1B

**FIGURE 10:**
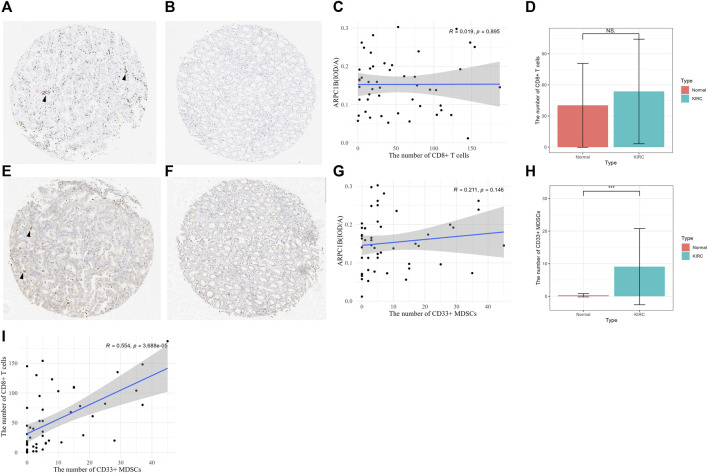
Relationship between CD8^+^ T cell and MDSCs and the expression of ARPC1B. **(A–B)** CD8^+^ T-cell expression was observed in KIRC and normal renal tissues (arrow indicates the clustering of CD8^+^ T cells). **(C)** Correlation between the number of CD8^+^ T cells and the expression of ARPC1B in KIRC. **(D)** Differences in the number of CD8^+^ T cells between KIRC and normal tissues. **(E–F)** CD33^+^ MDSCs expression observed in KIRC and normal renal tissue (arrow indicates the clustering of CD33^+^ MDSCs). **(G)** Correlation between the number of CD33^+^ MDSCs and the expression of ARPC1B in KIRC. **(H)** Differences in the number of CD33^+^ MDSCs between KIRC and normal tissues. **(I)** Correlation between the number of CD8^+^ T cells and number of CD33^+^ MDSCs in KIRC. NS (not significant with a *p*-value greater than 0.05), *p < 0.05, **p < 0.01, ***p < 0.001.

After excluding ineligible samples, a total of 49 cases of KIRC and 7 cases of normal tissues were included in the study. The number of CD8^+^ T cells was counted in KIRC (Figure 10A) and normal tissues (Figure 10B) on tissue chips. The results indicated a weak positive correlation between the number of CD8^+^ T cells and the expression of ARPC1B in KIRC, but there was no statistically significant difference (Figure 10C, *R* = 0.019, *p* = 0.895). Additionally, there was no difference observed in the number of CD8^+^ T cells between KIRC and normal tissues (Figure 10D, *p* > 0.05).

The number of CD33^+^ MDSCs was assessed in KIRC (Figure 10E) and normal tissues (Figure 10F) on tissue chips. The results showed that there was a positive correlation between the number of CD33^+^ MDSCs cells and ARPC1B expression in KIRC, but without statistical significance (Figure 10G, *R* = 0.211, *p* = 0.146). Moreover, the results demonstrated a difference in the number of CD33^+^ MDSCs between KIRC and normal tissues (Figure 10H, *p <* 0.001, Supplementary Figure S3). There was a positive correlation between the number of CD8^+^ T cells and the number of CD33^+^ MDSCs in KIRC, and this correlation was statistically significant (Figure 10I, *p* < 0.001).

## Conclusion

In this study, we analyzed the expression difference, clinical information, and prognostic value of ARPC1B in KIRC. First, to eliminate bias, we used TCGA, GEO, GEPIA, CPTAC, and UALCAN database analyses to conclude that the total mRNA and protein expression of ARPC1B was higher in KIRC than in normal tissues. In addition, qRT-PCR was used to confirm the high mRNA expression of ARPC1B in KIRC. However, IHC showed that there was no difference in the expression of ARPC1B between normal and KIRC tissues. Why was there a difference in total mRNA and protein between normal and KIRC tissues but no difference in IHC? The possible reason is that ARPC1B was specifically expressed in renal tubular cells, and only a part of cells express ARPC1B relative to the whole tissue. However, ARPC1B was diffusely expressed in most tumor tissues. The variations in ARPC1B expression levels per tissue volume unit lead to discrepancies in both total protein and mRNA levels. In normal renal tissues, only the expression of ARPC1B in renal tubule cells was observed by IHC, confirming that the Tabula Muris database describes the expression of ARPC1B in renal cells. Moreover, according to hints from the Tabula Muris database, ARPC1B may also be expressed in macrophages, leukocytes, and kidney capillary endothelial cells.

Then, we found that ARPC1B expression increased with increase in staging and grading, and lymph node metastasis and distant metastasis. Our findings indicate that a high expression of ARPC1B promotes tumorigenesis.

Our analysis reveals that a high expression of ARPC1B in KIRC is correlated with poor overall survival. Meanwhile, univariate and multivariate Cox analyses showed that the expression of ARPC1B is a useful biomarker for the prognosis of KIRC. These results suggest that ARPC1B is an independent prognostic factor of KIRC, and high expression of ARPC1B predicts poor prognosis.

We found that the main immune cells that positively correlated with ARPC1B expression were M0 macrophages, MDSCs, Tregs, Tfh, CD8^+^ T cells, CD8^+^ Tex, CD4^+^ T cells, Bm, NKa, CD56^+^ NK, and plasma cells. Of note, the results indicated that high ARPC1B expression in KIRC could significantly associate MDSCs and Tregs, while MDSCs and Tregs were reported to be immunosuppressive cells (De Cicco et al., 2020; Nishikawa and Koyama, 2021). They help tumors escape immune surveillance, contributing to tumor development and progression. The main activity of MDSCs was to inhibit the normal innate and adaptive immune function of immune cells. This mechanism was through inhibiting the activity of T cells and NK cells. In tumors, MDSCs have been proven to inhibit the proliferation and activation of cytotoxic T cells. The main mechanisms implicated in MDSC-mediated immune suppression include (ⅰ) deprivation of essential amino acids obtained by T cells; (ⅱ) decreased expression of L-selectin by T cells; (ⅲ) induction of oxidative stress; and (ⅳ) induction of immunosuppressive cells like Tregs and Th17 cells (Youn and Gabrilovich, 2010; Groth et al., 2019). In addition, MDSCs can also promote tumor angiogenesis and drug resistance. A large amount of evidence supported that the accumulation of MDSCs was closely related to the clinical outcome of cancer patients (Gabrilovich, 2017). Treg cells inhibit the activation of anti-tumor T cells by producing immunosuppressive cytokines and immunosuppressive CTLA4 (Nishikawa and Koyama, 2021). Zhang et al. suggested that Tregs were related to a worse prognosis in KIRC (Zhang et al., 2019). Current studies have found that MDSCs are potent inducers of Tregs, but Tregs can also regulate the differentiation and function of MDSCs (Fujimura et al., 2012).

TAM is a very important immune population that can promote or block the development of tumors (Murray et al., 2014). Interestingly, it was found that ARPC1B is positively correlated with M0 and negatively correlated with M1 and M2 macrophages. We hypothesized that the high expression of ARPC1B inhibited the further differentiation of M0 macrophages, thereby impairing their ability to fulfill their normal functions. A high fraction of M0 macrophages in KIRC was also associated with poor prognosis (Zhang et al., 2019).

The enrichment of GO annotation and pathway analysis showed that ARPC1B affected tumorigenesis and progression through multiple immune-related functions and pathways, such as regulation of immune effector process, regulation of inflammatory response, antigen processing and presentation, asthma, autoimmune thyroid disease, graft versus host disease, intestinal immune network for IgA production, and type I diabetic mellitus. Moreover, the results demonstrated that high ARPC1B expression had a positive correlation with multiple immune signatures (ssGSEA scores) such as CCR, checkpoint, cytolytic activity, HLA, inflammation-promoting, MHC class I, para-inflammation, TIL, and type I IFN responses. As mentioned earlier, the results suggested that ARPC1B was involved in the immune response of KIRC and played a role in immune escape.

To further confirm these findings, we selected and evaluated the correlation between eight important immune checkpoint genes and ARPC1B and utilized the TIDE algorithm to predict the response of high and low ARPC1B expressions to immune checkpoint blockade. The results discovered that SIGLEC15, CTLA4, HAVCR2, LAG3, PDCD1, and PDCD1LG2 were positively correlated with ARPC1B. However, the TIDE algorithm predicted that KIRC patients with a high expression of ARPC1B had a poorer response to immune checkpoint than those with a low expression of ARPC1B. These results confirmed that although high ARPC1B expression has a close relationship with some immune checkpoint genes, the response to immune checkpoint blockade was low. The TIDE algorithm suggested that the main reason for the poor response of the ARPC1B high-expression group to immune checkpoint blockade was T-cell dysfunction. The single-cell analysis of TISCH2 from KIRC_GSE111360 and KIRC_GSE121636 data sets showed that ARPC1B was positively correlated with CD8^+^ Tex. This means that T-cell exhaustion occurred in the ARPC1B high-expression group. This is consistent with our previous conclusion that high ARPC1B expression activates MDSCs and Tregs inhibits T-cell function and proliferation. In this case, patients lacked an effective response to ICB treatment. The results of this study are consistent with that reported in the literature (Law et al., 2020).

According to the literature report, anti-MDSC treatment and Tregs depletion can reactivate T cells (Colbeck et al., 2017; Fultang et al., 2019) and can greatly enhance the effect of ICB (Murray et al., 2014; Lee et al., 2020). Recently, it has been reported that targeting TAM and MDSC treatment enhances the blocking of PD-1 in cholangiocarcinoma (Loeuillard et al., 2020). This broadens the thinking of immunotherapy. Thus, high ARPC1B expression is considered to be highly correlated with MDSCs and Tregs, which may serve as a potential indicator for anti-MDSC treatment and/or Tregs depletion in KIRC. Although two small-molecule drugs targeting ARPC1B have been found, their pharmacological effects are still unclear.

The experimental results showed a statistically significant difference in the number of CD33^+^ MDSCs between KIRC and normal tissues, with a higher quantity observed in KIRC. These findings are consistent with the ssGSEA results. Interestingly, a positive correlation was observed between the numbers of CD8^+^ T cells and MDSCs in KIRC, indicating that the progression of KIRC may have stimulated the infiltration of CD8^+^ T cells into the tumor to eradicate cancer cells. However, the increased presence of MDSCs also exerts an immunosuppressive effect, which leads to functional impairment of CD8^+^ T cells. In our exploratory investigation into the relationship between CD8^+^ T cells or MDSCs and the expression of ARPC1B, our findings suggested a lack of substantial evidence supporting a significant correlation between these variables. The potential reasons for the discrepancy with the aforementioned conclusion could be attributed to the limitations of the immunocyte counting method in terms of its roughness and limited sensitivity. Additionally, tissue chips could have only represented partial information about the tissue and there might have been regional differences within the tissue that could have contributed to inconsistent counting results. Therefore, it is necessary to validate the presence of MDSCs and the expression of ARPC1B using alternative methods.

Of course, this study had some limitations. First, the sample size of the KIRC cohort study was limited, and more samples are required to obtain more accurate data. Second, the detailed mechanism of APRC1B expression and immune cell infiltration, specifically MDSCs and Tregs, requires further study. Furthermore, this study selected the median expression of ARPC1B as the cutoff value, which may not be optimal for further external data. Finally, the pharmacological effects of APRC1B-targeted drugs have to be further explored.

In conclusion, this study demonstrates that the high expression of ARPC1B is related to clinical progress, which is considered to be an independent risk factor for OS in patients with KIRC. The high expression of ARPC1B is closely related to the infiltration of MDSCs and Tregs. ARPC1B may affect the development and progression of the tumor by regulating tumor-infiltrating cells, MDSCs and Tregs, and play an important role in the immune escape of KIRC. This may help form a new treatment strategy and prolong the overall survival in KIRC patients.

## Data Availability

The original contributions presented in the study are included in the article/Supplementary Material; further inquiries can be directed to the corresponding author.
