# Supplementary material for: ARPC1B is a novel prognostic biomarker for kidney renal clear cell carcinoma and correlates with immune infiltration
Source: Front Mol Biosci. 2023 Sep 19;10:1202524. doi: 10.3389/fmolb.2023.1202524 (PMC10546172; doi:10.3389/fmolb.2023.1202524)

**Figure 1** The different mRNA and protein expressions of ARPC1B in KIRC and normal tissues. (**A**) ARPC1B mRNA expression levels in KIRC and normal tissues from TCGA and GTEx databases. (B) ARPC1B mRNA expression levels in KIRC and normal tissues in the GEPIA database. (C, D) Differential ARPC1B expression levels in KIRC and normal tissues from the GSE53757 and GSE66271 datasets. (E) The different total ARPC1B protein expression in KIRC and normal tissues from the CPTCA database. ^*^*P*<0.05, ^**^*P*<0.01, ^***^*P*<0.001.


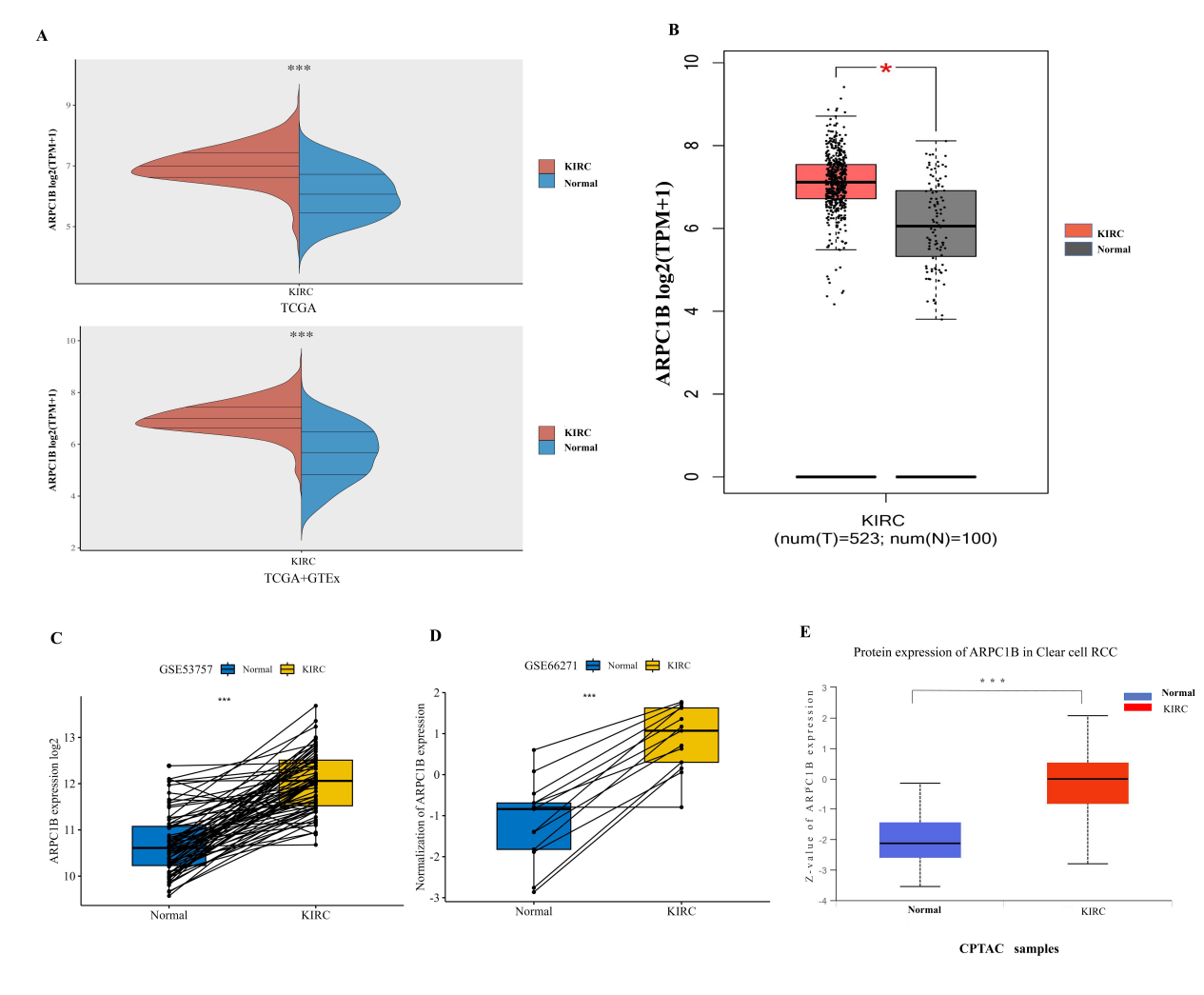


**Figure 2** The relationship between ARPC1B expression and clinicopathological characteristics in KIRC. (A-C) Relative mRNA expressions of ARPC1B in relation to gender, tumor stage, and tumor grade status. (D-F) Relative mRNA expressions of ARPC1B with respect to node metastasis, subtypes, and distant metastasis status.


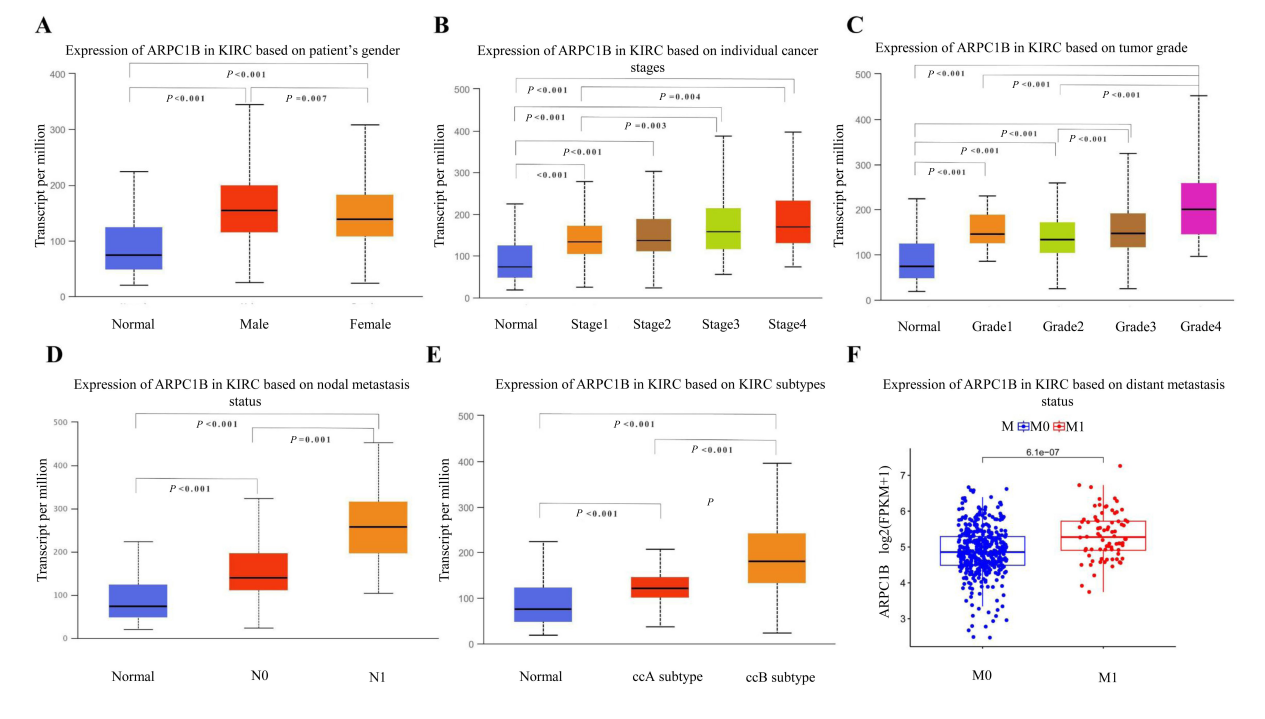


**Figure 3** The ROC curves illustrating the performance of ARPC1B expression in different scenarios. (A) ROC curves of ARPC1B expression in KIRC and normal tissues, (B) ROC curves comparing Stage Ⅰ/Ⅱ with Stage Ⅲ/Ⅳ, (C) ROC curves distinguishing between Grade 1/2 and Grade 3/4, (D) ROC curves for distinguishing metastasis M0 and M1.


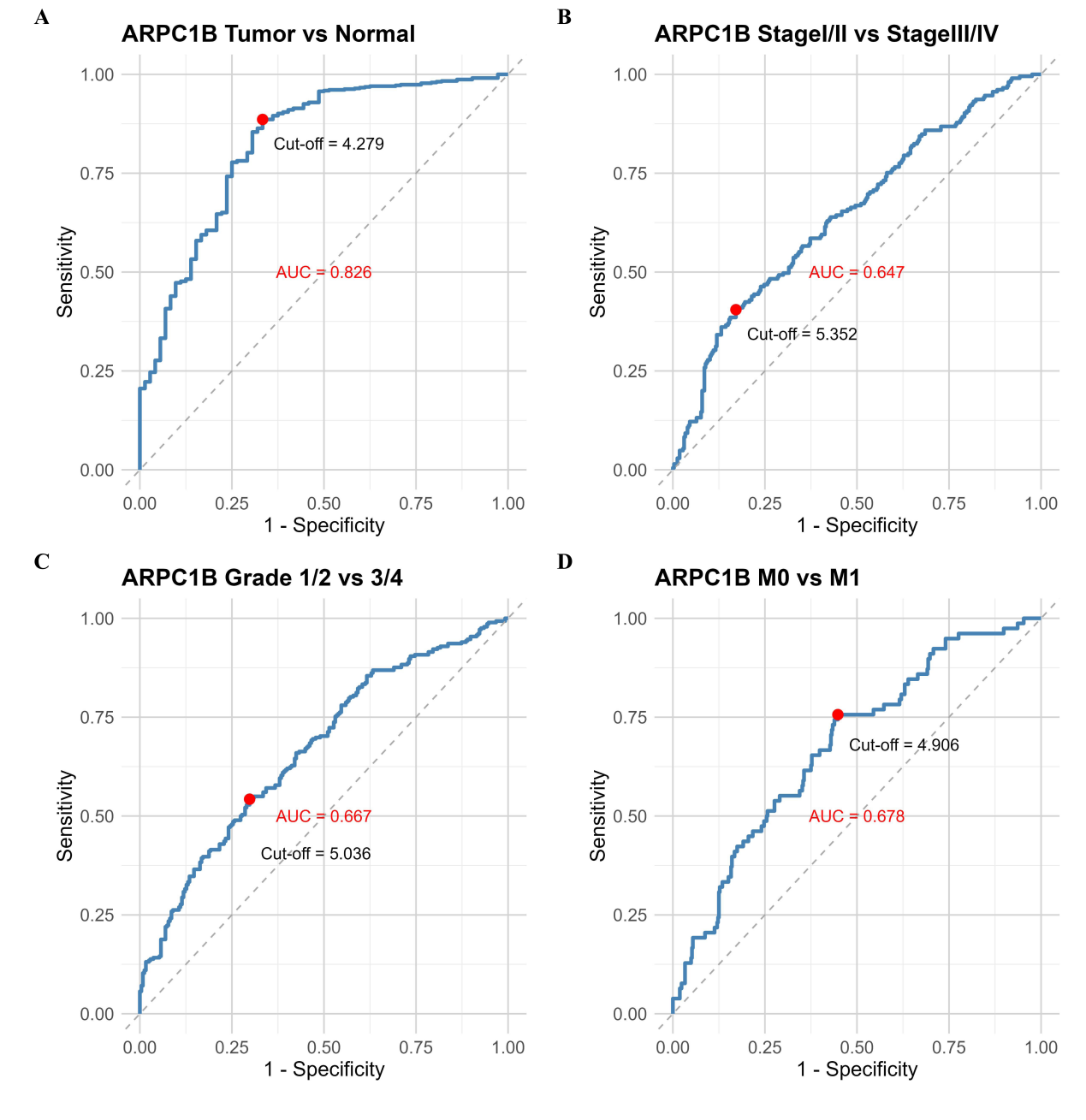


**Figure 4** Analysis of genomic alterations of ARPC1B in KIRC. (A) Genomic alterations of ARPC1B and corresponding dataset. (B) Types of ARPC1B gene alterations and their incidence. (C) Relationship between copy number variation and expression levels of ARPC1B.

**
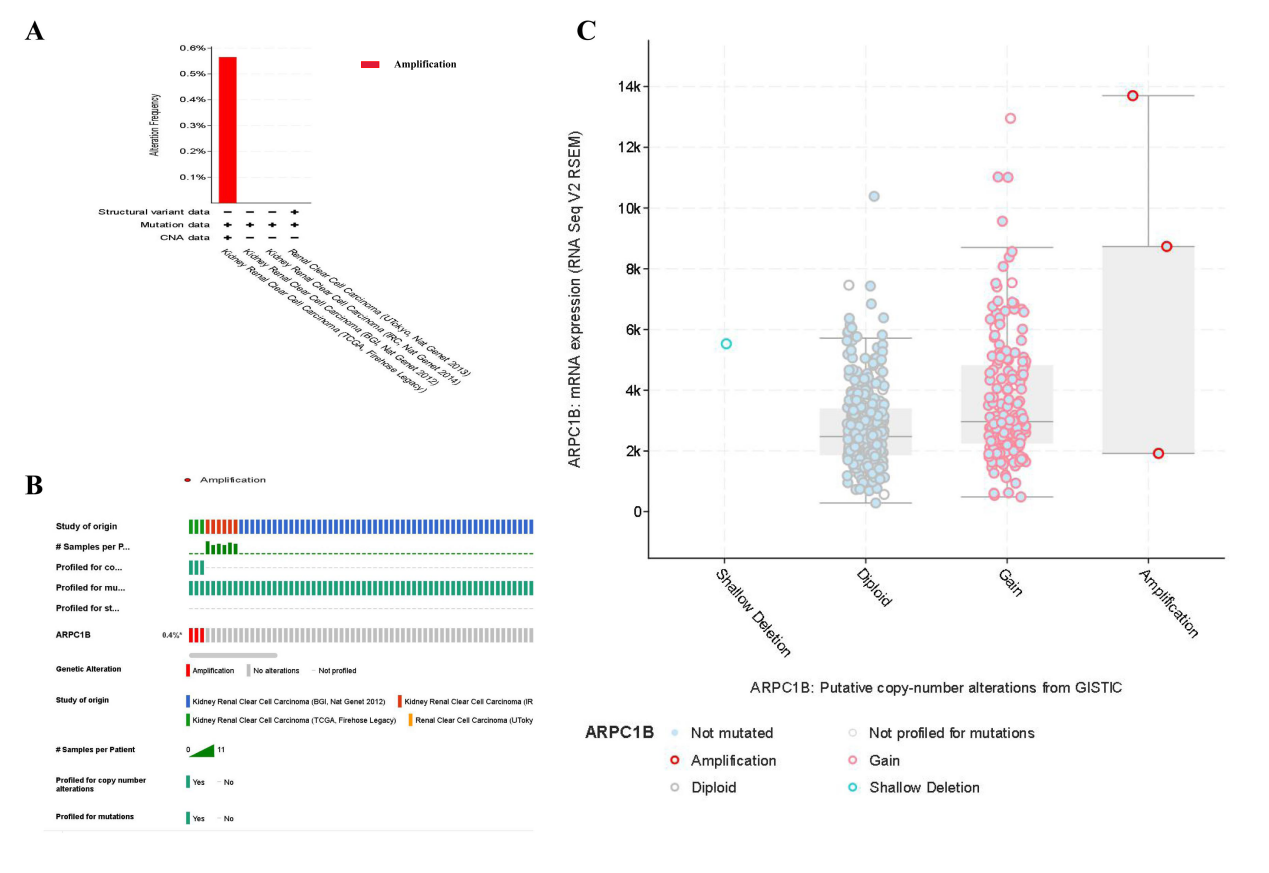
**

**Figure 5** Associations between ARPC1B expression and patient survival based on the TCGA dataset. (A) The top chart displayed patients sorted by risk scores and divided into high-risk and low-risk groups based on the median number of patients. The middle plot showed the relationship between survival time, survival state, and the high/low-risk groups. The bottom plot depicted the relationship between risk scores and normalized expression levels of ARPC1B. The bottom figure is a heatmap depicting the ARPC1B expression. (B) Kaplan-Meier survival analysis of ARPC1B expression, with comparisons among different groups made using the log-rank test. HR represents the hazard ratio of high-expression samples relative to low-expression samples. HR> 1 indicates that the gene is a risk factor, while HR<1 indicates that the gene is a protective factor. HR (95%CI) represents the hazard ratio along with its corresponding 95% confidence interval, as well as the median survival time (LT50) for different groups. (C)The ROC curve of the ARPC1B expression. The higher values of AUC correspond to a higher predictive power.


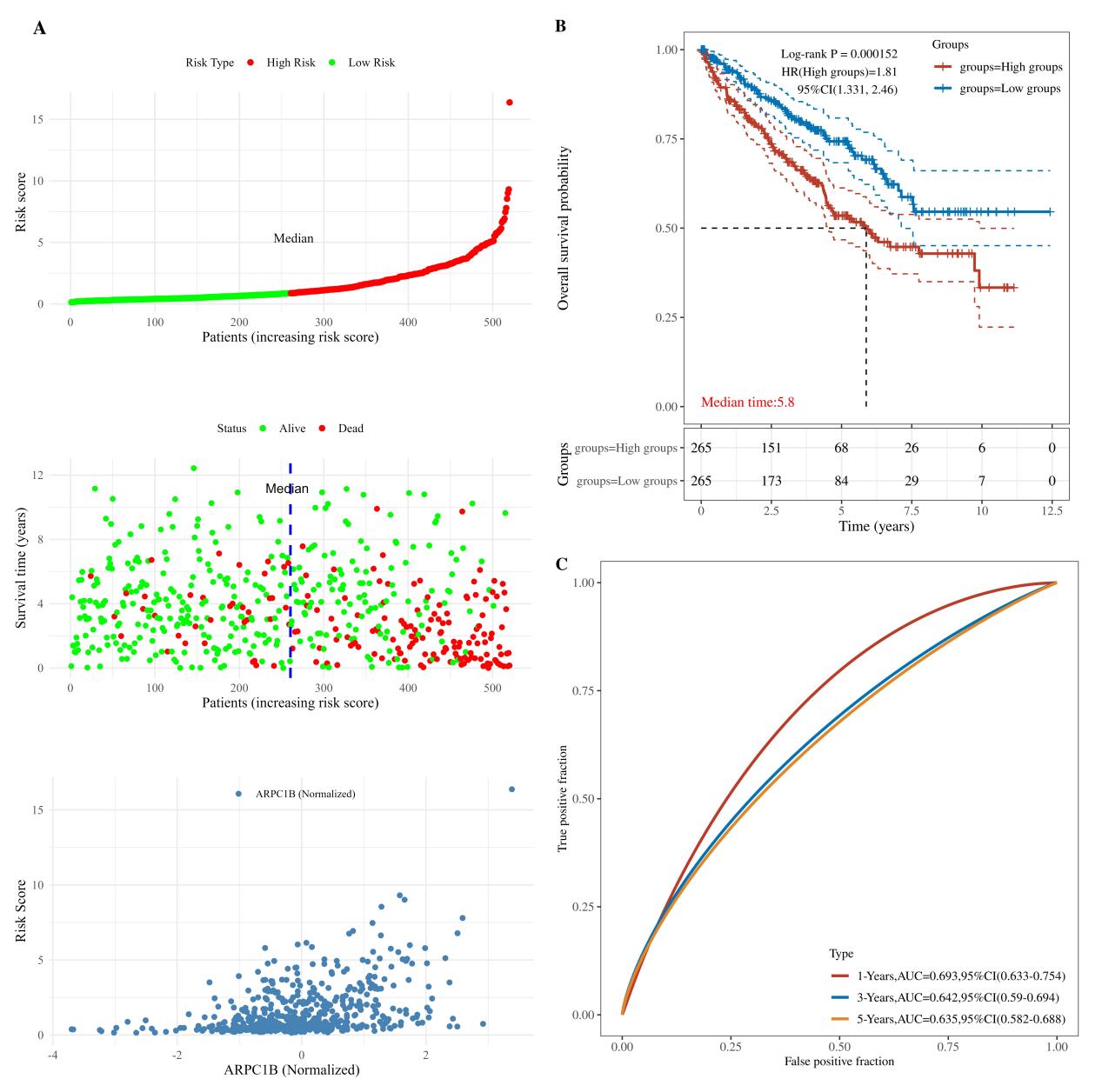


**Figure 6** Construction and evaluation of a prognostic model using the expression of ARPC1B and other clinical indicators in KIRC. (A) Univariate Cox regression analysis was performed to assess the statistical significance of ARPC1B and other clinical indicators, displaying the corresponding *P* value, HR, and 95%CI. (B) Multivariate Cox regression analysis was conducted to further elucidate the prognostic significance of ARPC1B and the selected clinical indicators. (C) Nomograms were developed to predict the 1-year, 3-year, and 5-year overall survival of KIRC patients based on the expression of ARPC1B and additional factors. (D) Calibration curve illustrating the performance of the overall survival nomogram model in the discovery group. The dashed diagonal line represents the ideal nomogram, while the blue, red, and orange lines represent the observed nomogram's predicted survival rates at 1-year, 3-year, and 5-year intervals.


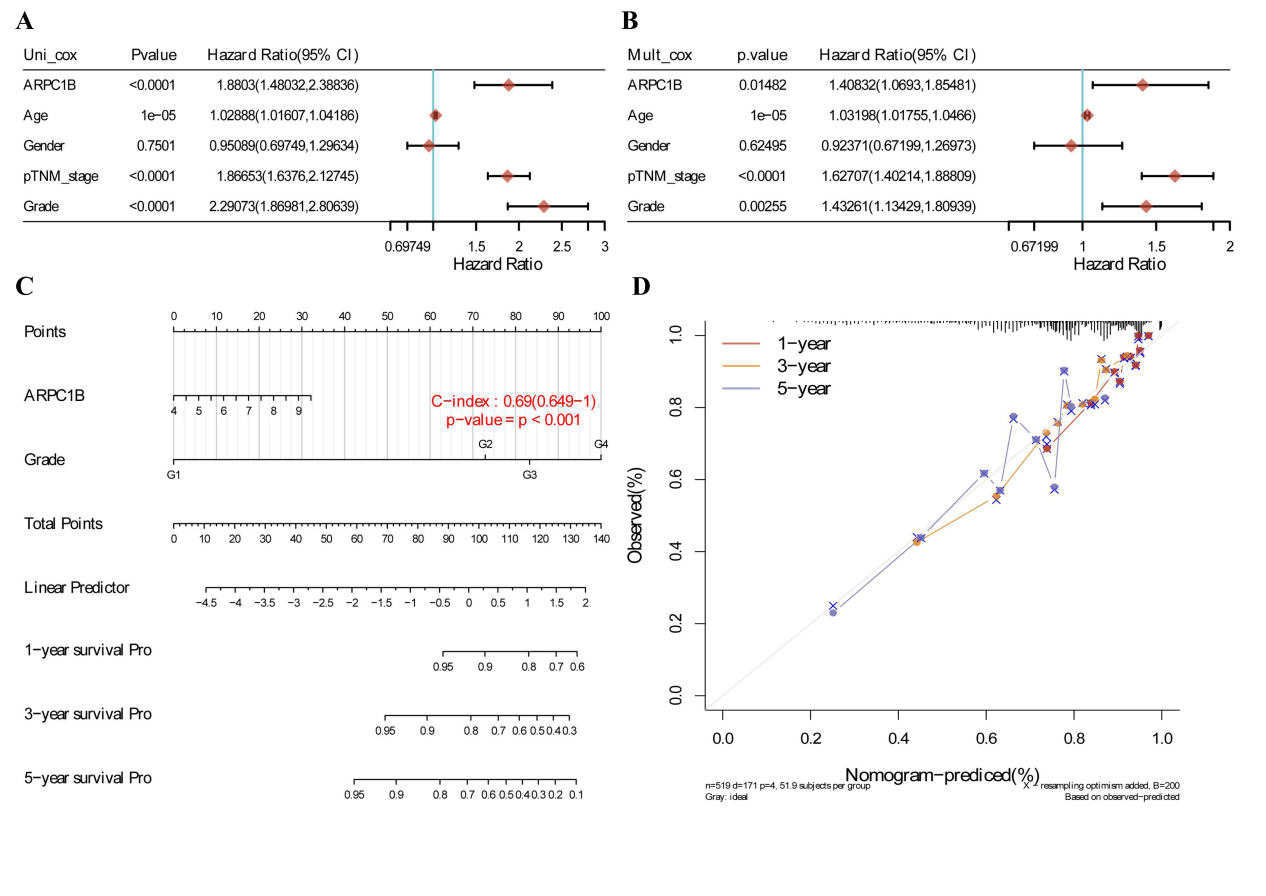


**Figure 7** Correlations between ARPC1B expression and tumor microenvironment, including immune cell infiltration, immune function, and immune subtypes in the KIRC. (A-C) The StromalScore (A), ImmuneScore (B), and ESTIMATEScore (C) were analyzed for their correlation with ARPC1B expression. The immune infiltration landscape was assessed using the CIBERSORT method (D), while ssGSEA analysis was employed to analyze immune cell infiltration in KIRC samples from the TCGA dataset (E). (F, G) TISCH2 analysis revealed the correlation between ARPC1B expression and immune cell populations. (H) ssGSEA analysis was utilized to examine Immune function. (I) The relationship between ARPC1B expression and immune subtypes was investigated.
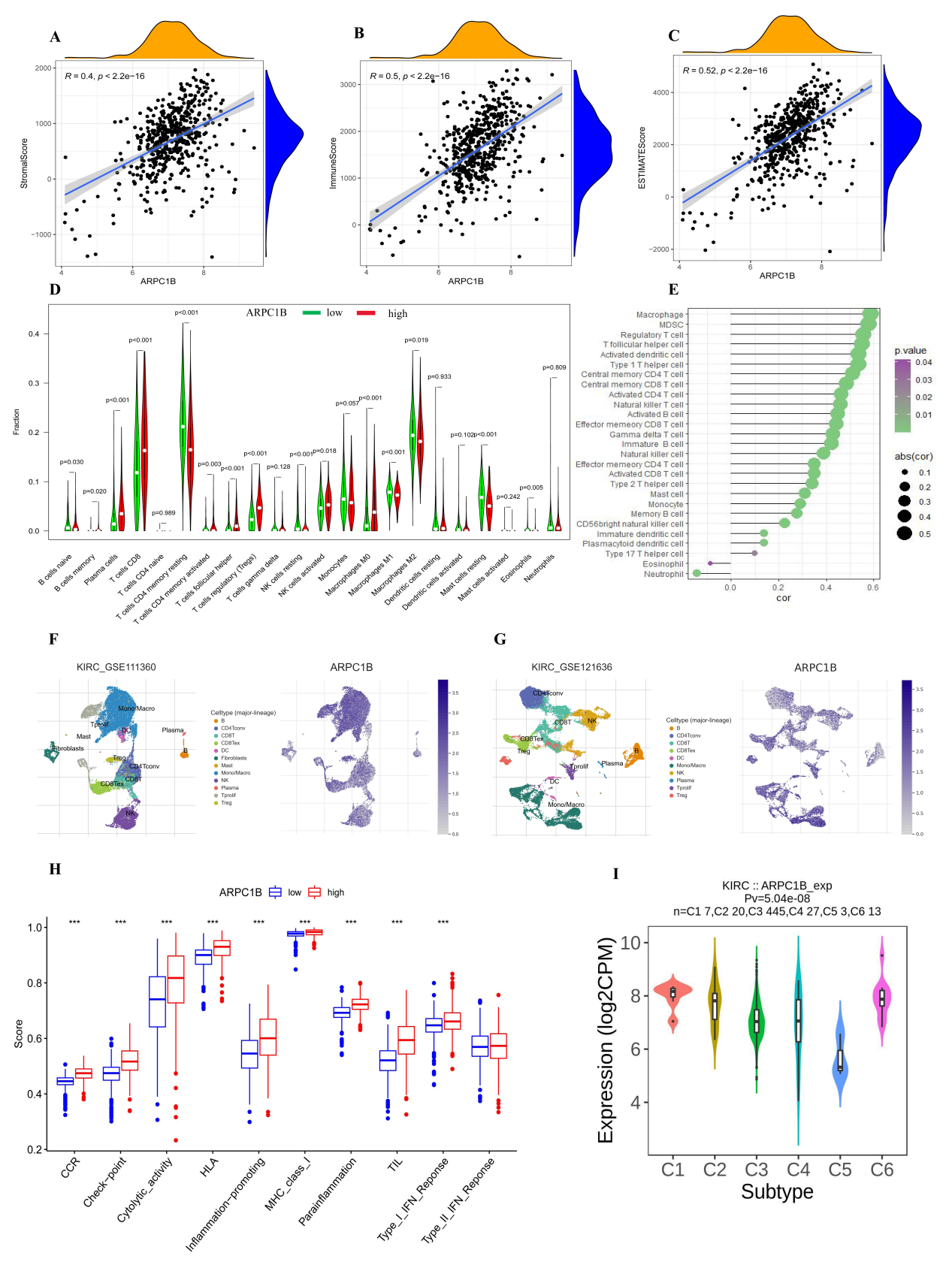


**Figure 8** Results of GO function annotation and KEGG path enrichment analysis, as well as responses to immune checkpoint blockade and their association with ARPC1B expression. (A) Enrichment analysis for GO function annotations. (B) Enrichment analysis for KEGG pathways based on ARPC1B expression. (C) GSEA analysis revealing the activation of signaling pathways associated with high APRC1B expression in KIRC. (D) Variation in TIDE scores between high and low APRC1B expression groups. (E) Differential responses to immune checkpoint blockade based on ARPC1B expression in KIRC. (F) Variation in T cell dysfunction between high and low APRC1B expression groups. *^*^P*<0.05, *^**^P*<0.01, ^***^*P*<0.001, ^****^*P*<0.0001.


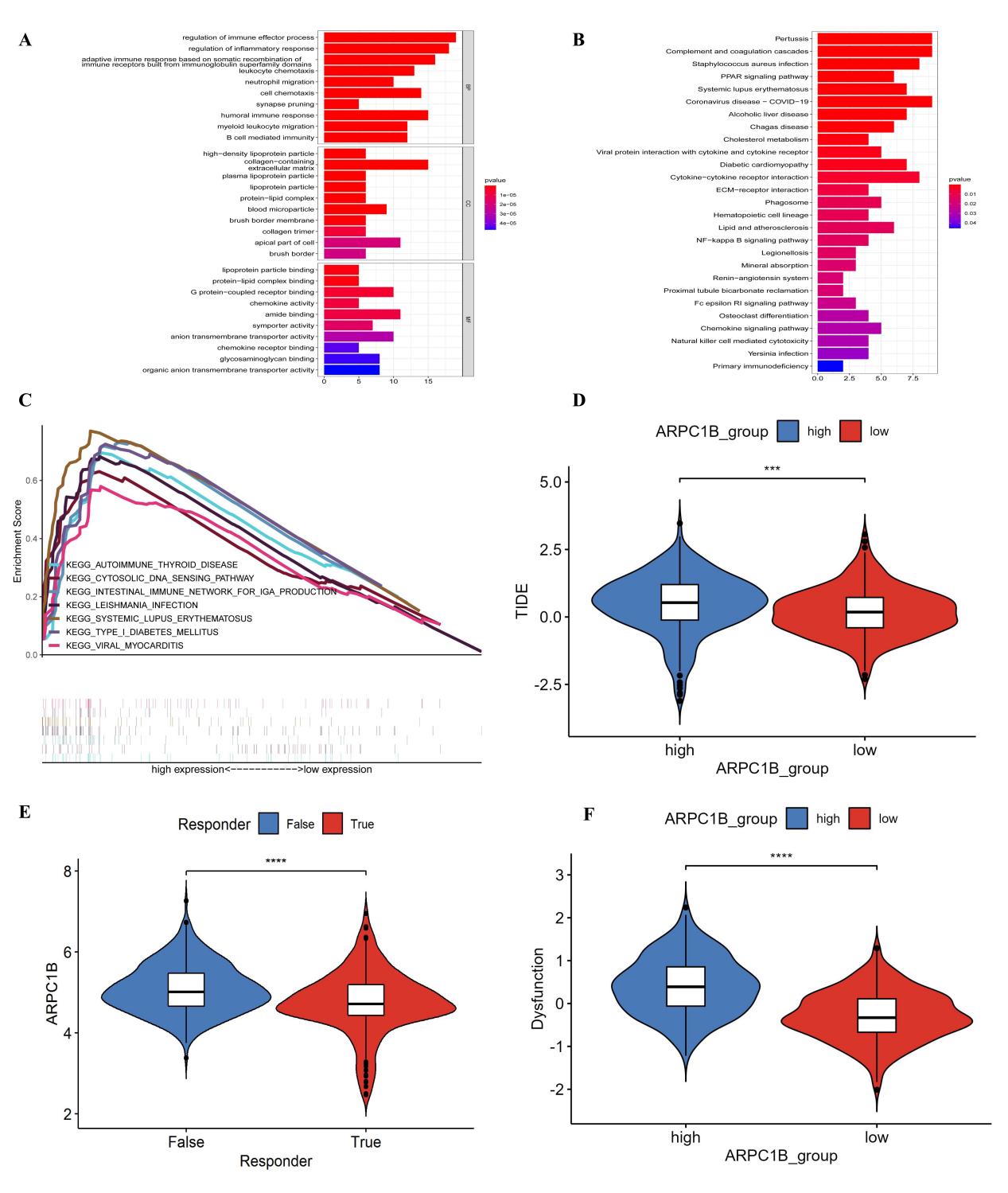


**Figure 9** Expression of ARPC1B in normal tissues and KIRC. (A) qRT-PCR analysis comparing ARPC1B expression between KIRC and normal tissue. (B-C) IHC images demonstrating ARPC1B expression in normal renal tissue (weak and strong expression). (E-G) IHC images depicting ARPC1B expression in KIRC with weak, medium, and strong expressions, respectively. The ratio of IOD to the area of IHC images is shown for comparisons between KIRC and normal tissue (D), Grade 1/2 versus Grade 3/4 (H), and Stage Ⅰ/Ⅱ versus Stage Ⅲ/Ⅳ (I). (J-K) ARPC1B expression in cell ontology classes using the Tabula database. NS (not significant with a *P*-value greater than 0.05), ^*^*P*<0.05, ^**^*P*<0.01, ^***^*P*<0.001.


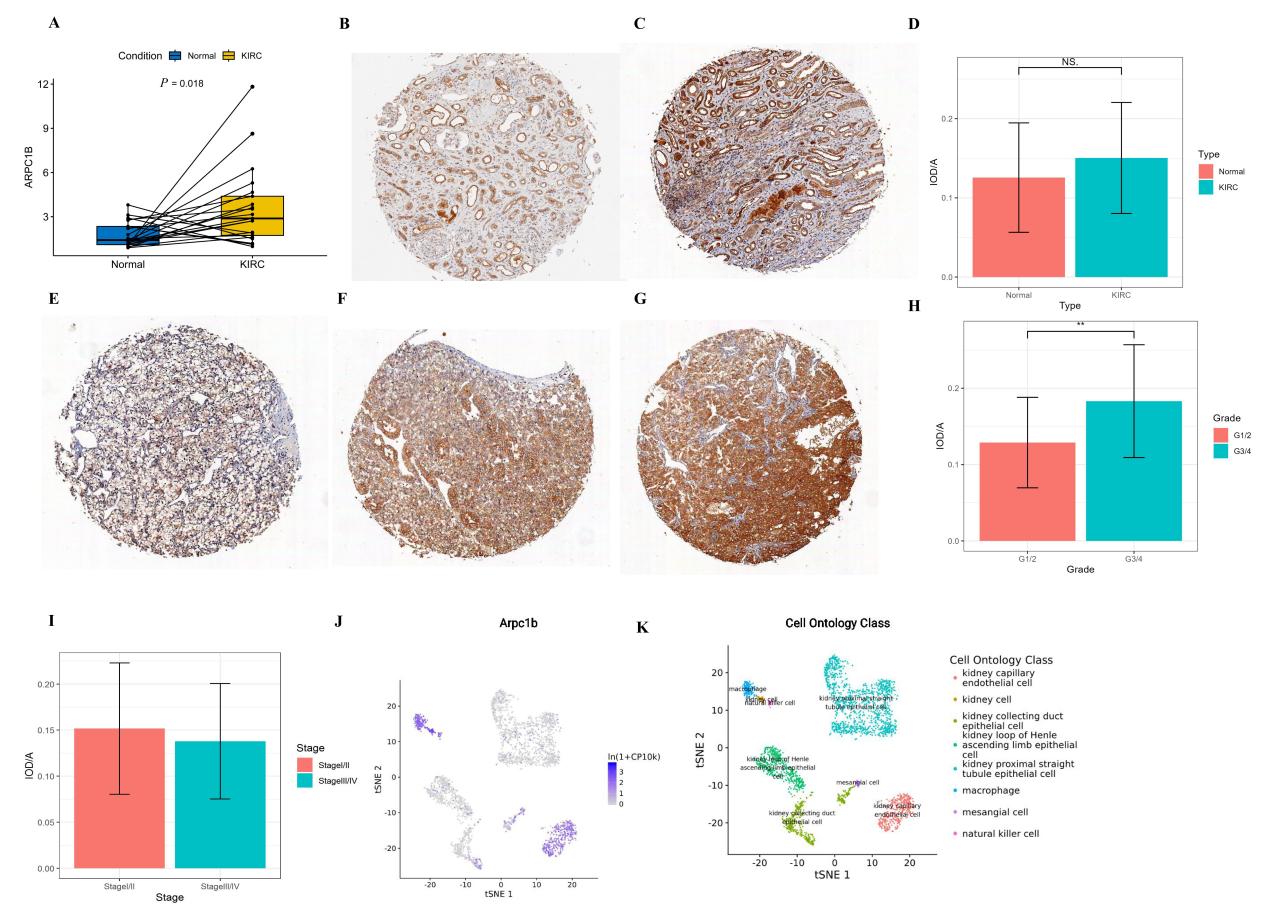


**Figure 10** The relationship between CD8+ T cell and MDSCs and the expression of ARPC1B. (A- B) CD8+ T cell expression was observed in KIRC and normal renal tissue (The arrow indicated the clustering of CD8+ T cells). (C) The correlation between the number of CD8+ T cells and the expression of ARPC1B in KIRC. (D) The differences in the number of CD8+ T cells between KIRC and normal tissues. (E-F) CD33+ MDSCs expression was observed in KIRC and normal renal tissue (The arrow indicated the clustering of CD33+ MDSCs). (G) The correlation between the number of CD33+ MDSCs and the expression of ARPC1B in KIRC. (H) The differences in the number of CD33+ MDSCs between KIRC and normal tissues. (I)The correlation between the number of CD8+ T cells and the number of CD33+ MDSCs in KIRC. NS (not significant with a *P*-value greater than 0.05), **P*<0.05, ***P*<0.01, ****P*<0.001.


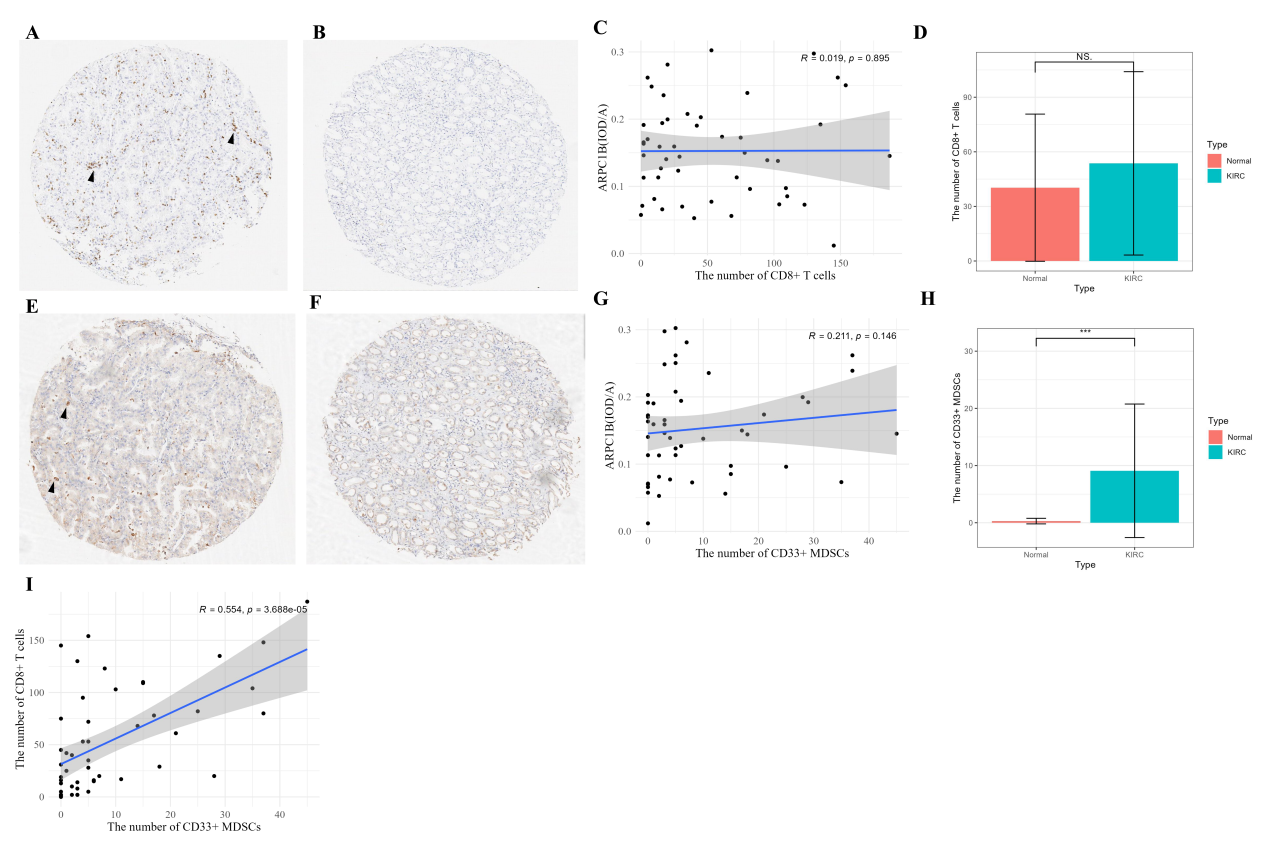


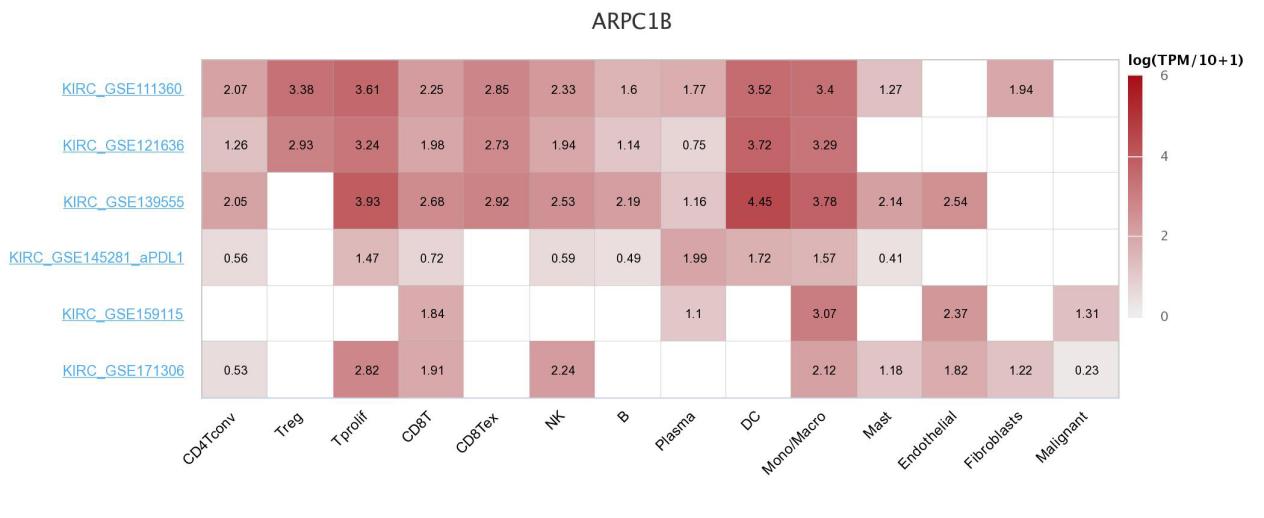
Supplementary **Figure 1** The heatmap depicting ARPC1B expression and immune cell populations from the TISCH database.

Supplementary **Figure 2** ARPC1B expression and cell ontology classification from the Tabula database.
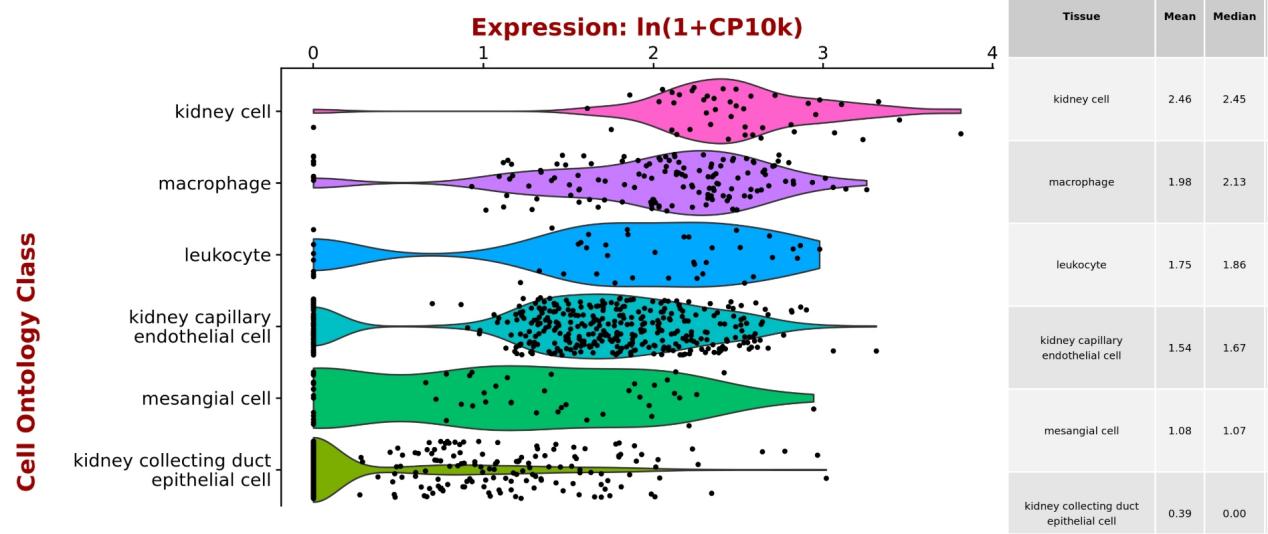


Supplementary **Figure 3** The differences in immune cells between KIRC and normal tissues using ssGSEA.


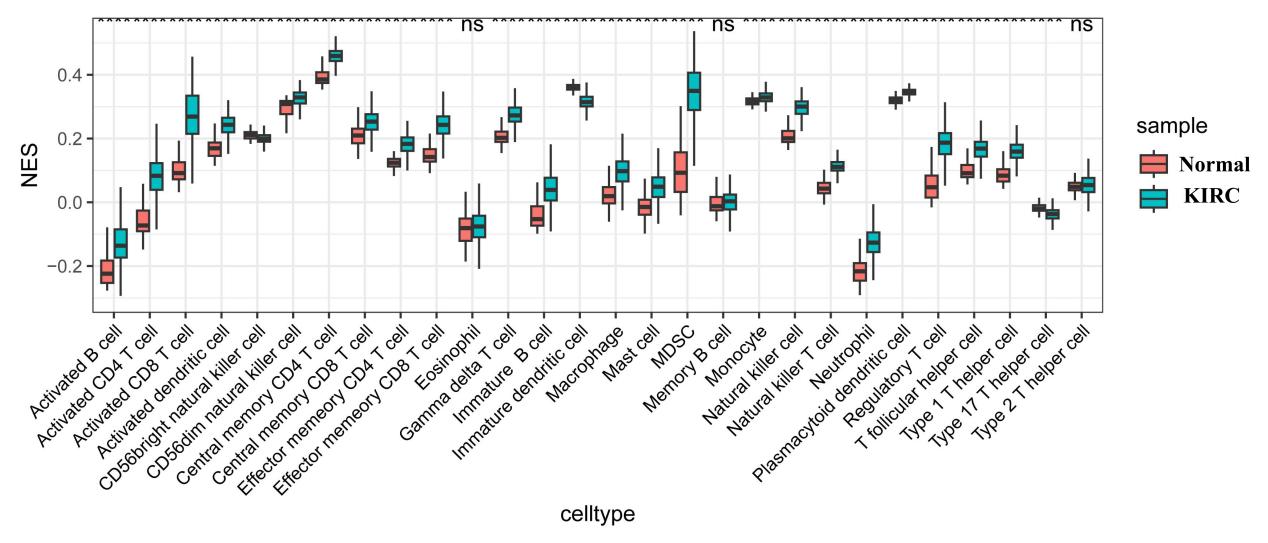

Supplement: Supplementary file 1 [file DataSheet1.docx]
